# Immune pressure is key to understanding observed patterns of respiratory virus evolution in prolonged infections

**DOI:** 10.1093/ve/veaf054

**Published:** 2025-07-21

**Authors:** Amber Coats, Yintong R Wang, Katia Koelle

**Affiliations:** Program in Microbiology and Molecular Genetics, Emory University, 1462 Clifton Road NE, Atlanta, GA 30322, United States; Department of Biology, Emory University, 1510 Clifton Road NE, Atlanta, GA 30322, United States; Department of Biology, Emory University, 1510 Clifton Road NE, Atlanta, GA 30322, United States; Emory Center of Excellence for Influenza Research and Response (CEIRR), Atlanta GA, United States

**Keywords:** prolonged viral infections, persistent infections, SARS-CoV-2, influenza A viruses, fitness landscape, within-host immune escape

## Abstract

Analyses of viral samples from prolonged SARS-CoV-2 infections as well as from prolonged infections with other respiratory viruses have indicated that there are several consistent patterns of evolution observed across these infections. These patterns include accelerated rates of nonsynonymous substitution, viral genetic diversification into distinct lineages, parallel substitutions across infected individuals, and heterogeneity in rates of antigenic evolution. Here, we use within-host model simulations to explore the drivers of these intrahost evolutionary patterns. Our simulations build on a tunably rugged fitness landscape model to first assess the role that mutations that impact only viral replicative fitness have in driving these patterns. We then further incorporate pleiotropic sites that jointly impact replicative fitness and antigenicity to assess the role that immune pressure has on these patterns. Through simulation, we find that the empirically observed patterns of viral evolution in prolonged infections cannot be robustly explained by viral populations evolving on replicative fitness landscapes alone. Instead, we find that immune pressure is needed to consistently reproduce the observed patterns. Moreover, our simulations show that the amount of antigenic change that occurs is higher when immune pressure is stronger and at intermediate immune breadth. While our simulation models were designed to shed light on drivers of viral evolution in prolonged infections with respiratory viruses that generally cause acute infection, their structure can be used to better understand viral evolution in other acutely infecting viruses such as noroviruses that can cause prolonged infection as well as viruses such as HIV that are known to chronically infect.

## Introduction

Many respiratory viruses, including influenza viruses and coronaviruses, typically cause acute infections that last less than two weeks (Bar-On et al., 2020, Einav et al., 2020). However, particularly in individuals who are immunocompromised or immunosuppressed, these viral infections can persist for much longer (Memoli et al., 2014, Baang et al., 2021, Dioverti et al., 2022, Machkovech et al., 2024). Sequencing of respiratory tract samples from individuals experiencing prolonged viral infections has revealed that a considerable amount of viral evolution can occur in these infections (Rogers et al., 2015, Xue et al., 2017, Borges et al., 2021, Chen et al., 2021, Kemp et al., 2021, Harari et al., 2022, Khatamzas et al., 2022, Ko et al., 2022, Nussenblatt et al., 2022, Quaranta et al., 2022, Scherer et al., 2022, Sonnleitner et al., 2022, Chaguza et al., 2023, Gonzalez-Reiche et al., 2023). Understanding these viral evolutionary dynamics is important for several reasons. First, these evolutionary dynamics can reveal whether the infecting virus has evolved to escape from a patient’s immune response or treatment regimen (McMinn et al., 1999, Rogers et al., 2015, Jensen et al., 2021, Kemp et al., 2021, Khatamzas et al., 2022, Scherer et al., 2022, Khosravi et al., 2023), and as such could help inform treatment strategies for the focal patient and more generally for individuals experiencing prolonged viral infections. Second, new viral variants that emerge at the host population level may originate from viruses that evolved in individuals experiencing prolonged infections, as has been discussed for SARS-CoV-2 (Berkhout and Herrera-Carrillo, 2022, Ghafari et al., 2022, Hill et al., 2022). Characterizing patterns of viral evolution within individuals with prolonged infections could therefore help in surveillance efforts at the level of the host population and efforts to anticipate phenotypes of forthcoming variants.

**Figure 1:**
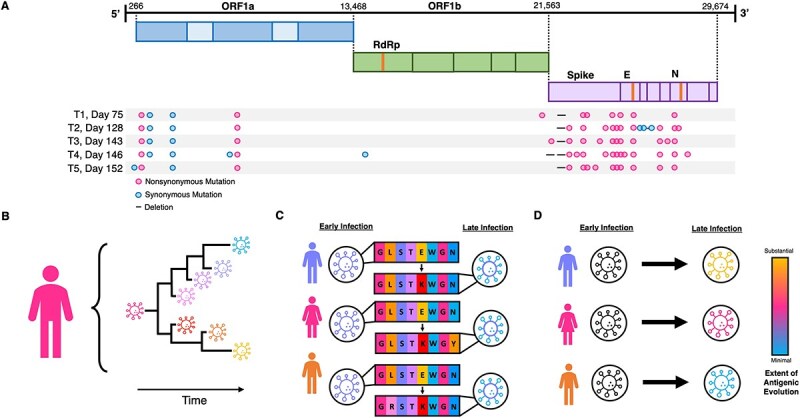
Evolutionary patterns of respiratory viruses observed in individuals with prolonged infection. (A) Nonsynonymous substitution rates generally exceed synonymous substitution rates. The panel shows nonsynonymous and synonymous viral substitutions that have accrued in an individual experiencing a prolonged SARS-CoV-2 infection. Sample days and substitutions are relative to the individual’s T0, Day 0 consensus sequence. Schematic reproduced from Choi et al. (2020) with permission from the Massachusetts Medical Society/New England Journal of Medicine. (B) Multiple distinct viral lineages often evolve within individuals with prolonged infections. (C) Parallel substitutions often arise across individuals with prolonged infection. The schematic shows consensus viral sequences from early and late infection timepoints of three infected individuals. Arrows highlight the frequently observed E484K substitution in the spike gene of SARS-CoV-2. (D) Rates of antigenic evolution are variable across individuals with prolonged infection. The schematic depicts heterogeneity in the extent of antigenic evolution that occurs over time in three individuals.

Many studies have described patterns of respiratory virus evolution within individuals with prolonged infections (Rocha et al., 1991, Rogers et al., 2015, Xue et al., 2017, Borges et al., 2021, Chen et al., 2021, Kemp et al., 2021, Harari et al., 2022, Khatamzas et al., 2022, Ko et al., 2022, Nussenblatt et al., 2022, Quaranta et al., 2022, Riddell et al., 2022, Scherer et al., 2022, Sonnleitner et al., 2022, Wilkinson et al., 2022, Chaguza et al., 2023, Gonzalez-Reiche et al., 2023, Rutsinsky et al., 2024). In these studies and others, four evolutionary patterns are frequently observed: (I) Nonsynonymous substitution rates tend to be higher than synonymous substitution rates (Choi et al., 2020, Harari et al., 2022, Chaguza et al., 2023, Markov et al., 2023) (Fig. 1A), particularly in viral genes that code for surface proteins; (II) Multiple co-circulating viral lineages often establish within individuals with prolonged infection (Rogers et al., 2015, Chaguza et al., 2023, Machkovech et al., 2024) (Fig. 1B); (III) Parallel viral substitutions often occur across individuals experiencing prolonged infection (Memoli et al., 2014, Xue et al., 2017, Wilkinson et al., 2022) (Fig. 1C); and (IV) The extent of antigenic evolution that is observed across individuals with prolonged infection is highly variable (Rocha et al., 1991, McMinn et al., 1999, Xue et al., 2017, Harari et al., 2022) (Fig. 1D).

While these four patterns of respiratory virus evolution in individuals with prolonged infection are well established, we still lack a comprehensive understanding of their drivers. Here, we develop a simulation model for respiratory virus evolution within individuals with prolonged infection and simulate this model under various parameterizations to help shed light on possible drivers of within-host viral evolution in these longer-term infections. Specifically, we extend a tunably rugged fitness landscape model (Aita et al., 2000, Neidhart et al., 2014) to consider viral evolution at sites that are either synonymous or nonsynonymous, with the latter impacting either viral replicative fitness, antigenicity, or both replicative fitness and antigenicity (sites that we call pleiotropic sites). We simulate this model to identify features of the viral fitness landscape and characteristics of the immune response that can reproduce these four evolutionary patterns that have been empirically observed in prolonged respiratory virus infections. From these simulations, we find that immune pressure is key to consistently reproducing all four viral evolutionary patterns shown in Fig. 1.

## Methods

### The fitness landscape model

**Figure 2:**
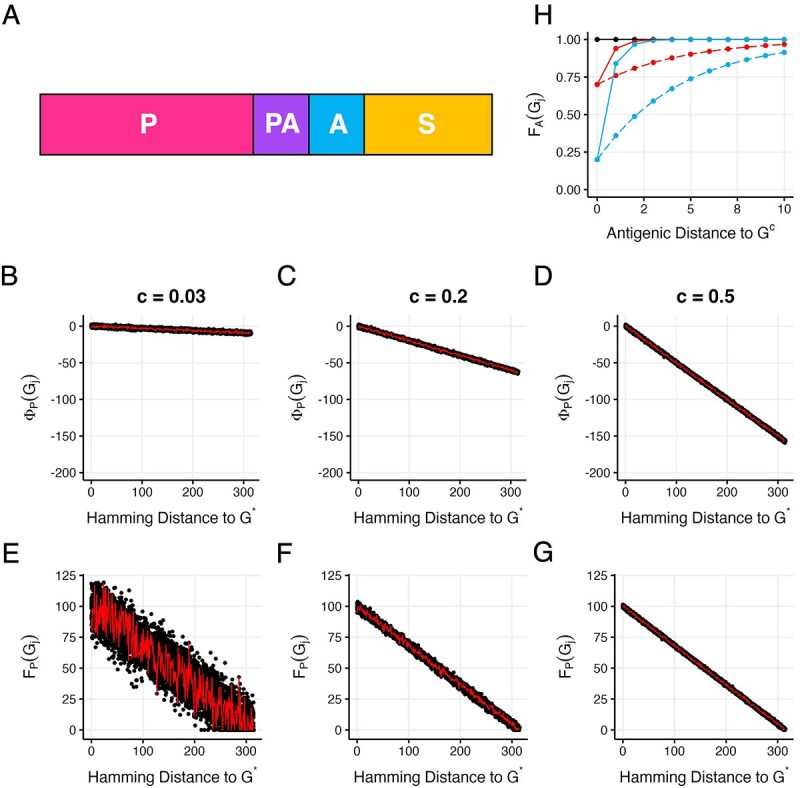
Structure of the viral genome and projections of replicative fitness and antigenic fitness. (A) Structure of the viral genome, showing the 4 different types of sites: phenotypic ($P$), pleiotropic ($PA$), antigenic ($A$), and synonymous ($S$). Site order is not important because we do not consider the process of recombination in our simulations of viral evolution. (B) Projection of the RMF fitness landscape given by equation (1), parameterized with a small $c$ to implement a highly rugged fitness landscape. Here, $c = 0.03$. (C) Projection of the RMF fitness landscape given by equation (1), parameterized with a $c$ of 0.2 to implement a semi-rugged fitness landscape. (D) Projection of the RMF fitness landscape with a large $c$ to implement a relatively smooth fitness landscape. Here, $c=0.5$. In (B–D), $G^{*}$ is given by the bitstring of all ones and the genome length is set to $L_{P} + L_{PA} = 315$. (E–G) Linearly transformed fitness landscapes, derived from panels (B–D), with parameter $k$ set to 100. The red lines in panels (B-G) show one genetic pathway consisting of single point mutations from the bitstring of all ones ($G^{*}$; Hamming distance to $G^{*} = 0$) to the bitstring of all zeros (Hamming distance to $G^{*} = 315$). H) Antigenic fitness of genotypes that are different antigenic distances away from the consensus genotype $G^{c}$. The black line shows the antigenic fitness function parameterized with $q=0$ (and any value of $p$ between 0 and 1). The red line shows $F_{A}$ with $q=0.3$ and $p = 0.2$, corresponding to immune strength being weak and immune breadth being narrow. The red dashed line shows $F_{A}$ with $q=0.3$ and $p = 0.8$, corresponding to immune strength being weak and immune breadth being moderate. The blue line shows $F_{A}$ with $q=0.8$ and $p = 0.2$, corresponding to immune strength being strong and immune breadth being narrow. The blue dashed line shows $F_{A}$ with $q=0.8$ and $p = 0.8$, corresponding to immune strength being strong and immune breadth being moderate.

We model the viral genome as consisting of four different types of sites: synonymous sites ($S$), phenotypic sites ($P$), antigenic sites ($A$), and pleiotropic sites ($PA$) (Fig. 2A). Mutations falling on synonymous sites are assumed to be silent and do not impact viral fitness. Mutations falling on phenotypic sites are nonsynonymous and impact phenotypes related to replicative fitness. Mutations falling on antigenic sites are nonsynonymous and impact only antigenicity. Mutations falling on pleiotropic sites are nonsynonymous and simultaneously impact both replicative fitness and antigenicity. The number of sites in each of these four classes is given by $L_{S}$, $L_{P}$, $L_{A}$, and $L_{PA}$, respectively, with the total number of sites in the viral genome given by $L = L_{S} + L_{P} + L_{A} + L_{PA}$. Genotypes are modeled as bitstrings, with each site carrying one of two possible alleles: 0 or 1. As such, genotype space consists of $2^{L}$ genotypes. We determine the overall fitness of a given viral strain by taking into consideration its replicative fitness as well as its antigenic phenotype.

To quantify the replicative fitness of a viral genotype, we extend an existing fitness landscape model called the Rough Mount Fuji (RMF) model (Aita et al., 2000, Neidhart et al., 2014). The original model has two parameters: the length of the genome and a parameter $c$ that controls the ruggedness of the landscape. The landscape is a static landscape, with the fitness of a genotype that does not change over time. We thus use the RMF landscape only for quantifying the replicative fitness component of a viral genotype. Because in our model replicative fitness depends only on the alleles present at $P$ and $PA$ sites, the length of the genome we use for the RMF model is given by $L_{P} + L_{PA}$, resulting in a total of $2^{(L_{P} + L_{PA})}$ possible genotypes on the landscape. Fitness values in the RMF model are initially calculated according to the following equation (Neidhart et al., 2014):


(1) \begin{align*}& \Phi_{P}(G_{j}) = -cD(G_{j},\ G^{*}) + \eta(G_{j})\end{align*}

where $G_{j}$ denotes focal genotype $j$ and $G^{*}$ denotes a reference genotype. The first term, $-cD(G_{j},\ G^{*})$, is a product of the non-negative parameter $c$ and the Hamming distance $D$ between genotype $j$ and reference genotype $G^{*}$. The Hamming distance is defined as the number of sites at which the two genotypes differ. The minimum value of $D$ is 0, which occurs when $G_{j} = G^{*}$. The maximum value of $D$ is $L_{P} + L_{PA}$, which occurs when focal genotype $G_{j}$ differs from $G^{*}$ at every site that contributes to replicative fitness. The second term, $\eta (G_{j})$, is a discrete random variable that is drawn from a specified probability distribution function. Here, we use a Gaussian distribution with mean 0 and standard deviation 1 to generate our $\eta $ random variables. This RMF model generates a tunably rugged fitness landscape model, with the random variable $\eta (G_{j})$ contributing the ruggedness and the parameter $c$ modulating the extent of the ruggedness. When $c = 0$, the fitness landscape reduces to a House of Cards landscape, where the fitness value of a genotype is uncorrelated with the fitness value of genotypes that surround it. At low $c$ (high ruggedness), epistatic interactions dominate. As $c$ gets larger, epistatic interactions become weaker and the fitness landscape becomes less rugged, reducing to a smooth Mount Fuji landscape as $c \rightarrow \infty $, with genotype $G^{*}$ being the genotype with highest fitness. Figure 2B–D project the RMF landscape for three different values of $c$: $c = 0.03$, $c = 0.2$, and $c = 0.5$.

Under this model, the range of values that the fitness landscape spans differs depending on the value of $c$ chosen. When $c$ is larger, the range of fitness values is larger (Fig. 2B–D). To be able to compare patterns of viral evolution across fitness landscapes of different ruggedness, we therefore modified the above RMF model such that the overall distribution of fitness values would be similar across $c$ values. We did this by linearly transforming the $\Phi _{P}$ fitness values so that they fall between 0 and $k$, where $k$ is a non-negative parameter, similar to the approach taken in Greene and Crona (2014). We denote the linearly-transformed replicative fitness value of genotype $G_{j}$ as $F_{P}(G_{j})$. The equation used for linear transformation is: $F_{P}(G_{j}) = (m \times \Phi _{P}(G_{j})) + b$ where the slope $m = k/(c \times (L_{P} + L_{PA}))$ and the y-intercept $b = k$. Because linear transformation may result in some genotypes (particularly those that are distant from the reference genotype) having negative fitness values, we further set the fitness values $F_{P}$ of all genotypes with negative fitness values to 0. Fig. 2E–G show the linearly-transformed fitness landscapes for the RMF landscapes shown in Fig. 2B–D, respectively, with $k$ set to 100. These linearly-transformed fitness landscape projections demonstrate that a lower value of the parameter $c$ results in a more rugged fitness landscape, with many local fitness peaks present across the landscape.

We allow antigenic changes from mutations occurring at either antigenic ($A$) sites or pleiotropic ($PA$) sites to impact overall viral fitness. Specifically, we assume that the overall fitness of a viral genotype $G_{j}$ depends multiplicatively on its replicative fitness and its antigenic fitness:


(2) \begin{align*}& F(G_{j}) = F_{P}(G_{j}) \times F_{A}(G_{j}).\end{align*}

Here, antigenic fitness $F_{A}(G_{j})$ is a value that lies between 0 and 1. When $F_{A}(G_{j})$ is closer to 0, there is substantial immune pressure against genotype $G_{j}$ that results in its overall fitness being close to 0. As $F_{A}(G_{j})$ approaches 1, immune pressure against genotype $G_{j}$ is weaker and overall fitness is determined solely by replicative fitness. By modeling antigenic fitness in this manner, we are assuming that it modulates viral fitness by introducing a fitness cost. We model antigenic fitness using the function:


(3) \begin{align*}& F_{A}(G_{j}) = 1- q \times p^{D(G_{j}(a), G^{c}(a))}\end{align*}

where $G^{c}$ denotes the consensus genotype circulating in the viral population and $D(G_{j}(a), G^{c}(a))$ denotes the Hamming distance between genotype $G_{j}$ and consensus genotype $G^{c}$ at exclusively antigenic and pleiotropic sites (notationally referred to here as $a$ sites). This function contains 2 parameters. Taking on values between 0 and 1, parameter $q$ can be thought of as a parameter that modifies the *strength* of immune pressure. When $q = 0$, the antigenic fitness of the consensus genotype (and all genotypes) is 1, reflecting an absence of immune pressure. As $q$ approaches 1, the antigenic fitness of the consensus genotype approaches 0, indicating that immune pressure is strong and considerably impacts overall viral fitness. Parameter $p$ can be thought of as a parameter that modifies the *breadth* of immunity. Also taking on values between 0 and 1, when $p$ is closer to 0, the breadth of the immunity is narrow, with genotypes that are only a single antigenic mutation away from the consensus genotype experiencing only very weak immune pressure (their $F_{A}$ values are close to 1). As $p$ approaches 1, immune protection becomes broader, with genotypes that are several antigenic mutations away from the consensus genotype still experiencing similar immune pressure to that of the consensus genotype. Figure 2H shows the antigenic fitness values of viral genotypes that are different antigenic distances away from the consensus genotype under different parameterizations of $q$ and $p$.

A mutation at a synonymous ($S$) site that generates viral genotype $G_{j}$ does not impact either $F_{P}(G_{j})$ or $F_{A}(G_{j})$, such that the overall fitness of the mutant is the same as its parent. A mutation at a phenotypic ($P$) site impacts only $F_{P}(G_{j})$. A mutation at an antigenic ($A$) site impacts only $F_{A}(G_{j})$. A mutation at a pleiotropic ($PA$) site impacts both $F_{P}(G_{j})$ and $F_{A}(G_{j})$. Mutations at both $P$ and $PA$ sites impact replicative fitness on the RMF landscape and thus interact epistatically with other $P$ and $PA$ sites. The formulation of equation (2) allows for a trade-off between replicative fitness $F_{P}$ and immune escape (captured by $F_{A}$) in that a mutation at a pleiotropic site may result in a viral genotype that is further away from the consensus genotype (thus enabling immune escape and increasing antigenic fitness $F_{A}$) and at the same time result in lower replicative fitness $F_{P}$. However, the formulation also allows for a mutation to simultaneously enable immune escape while increasing replicative fitness. This would occur if the mutation increases both $F_{A}$ and $F_{P}$. Our model formulation thus does not *impose* a trade-off between replicative fitness and immune escape, but it *allows* for such a trade-off to occur. One would expect a trade-off to occur more frequently when the virus is already well-adapted to the host simply because a mutation impacting replicative fitness would have a higher chance of being deleterious if the virus was already well-adapted. Finally, it is important to note that, by the formulation of equation (3), we are assuming that immune escape is fleeting in that viral fitness with respect to antigenicity only depends on how different a genotype is from the consensus genotype at the present time and not the history of viral genotypes that have circulated over the course of infection.

### Simulating within-host viral evolution

We simulate within-host viral evolution under the assumption of a fixed viral population size $N$ using Gillespie’s $\tau $-leap algorithm (Gillespie, 2001). Initially, at the time of infection ($t = 0$), each viral particle is set to the same infecting genotype. We adopt this assumption to reflect the low levels of viral diversity at the start of infection that result from a tight transmission bottleneck (McCrone et al., 2018, Lythgoe et al., 2021, Martin and Koelle, 2021, Shi et al., 2024). We then update the evolving viral population from one time point to the next by determining which viral particles will die and which viral particles will reproduce over the next $\Delta t$ time increment. To determine which viral particles will die, we first draw a random number $n$ from a Poisson distribution with mean $d N \Delta t$, where $d$ is the per capita viral death rate. We then choose $n$ viral particles at random and without replacement to be removed from the viral population. To determine which viral particles will reproduce, we draw $n$ viral particles (with replacement) in proportion to their overall fitness values $F$. Progeny viruses inherit the genotype of their respective parents, plus any additional mutations that occur during their “birth.” The number of additional mutations that occur during the birth of a given viral particle is drawn from a Poisson distribution with mean $\mu L$, where $\mu $ is the per site per infection cycle mutation rate and $L$ is the total number of sites in the viral genome. A mutation results in a flip of the parental allele (from either 0 to 1 or 1 to 0). The sites at which mutations occur are selected at random from the viral genome. Once viral particle deaths and births have been simultaneously updated, time is incremented from $t$ to $t + \Delta t$.

When viral genome sizes are very small, it is possible to calculate the replicative fitness values of each genotype prior to simulating viral evolution on the landscape. However, even with only 100 sites that impact replicative fitness, genotype space becomes too large to adopt this approach. When simulating viral evolution, we therefore dynamically allocate $F_{P}$ fitness values as strains are accessed through mutation over the course of a prolonged infection. Adopting this approach allows us to calculate and store a considerably smaller portion of viral genotype space.

### Model parameterization

We simulated intrahost viral evolution using viral genomes that have a total of either $L=400$ or $L=800$ sites. Our choice of these genome sizes attempted to strike a balance between computational tractability and the $\sim $660 base pair lengths of the receptor binding domains of SARS-CoV-2 and influenza A viruses. In our $L=400$ simulations, we let 315 of these sites be nonsynonymous and the remaining 85 sites be synonymous ($S$). This ratio of 315:85 reflects the approximate ratio of 3.7 in nonsynonymous to synonymous sites observed for SARS-CoV-2 and influenza viruses. Of the 315 nonsynonymous sites, we consider in our simulations different scenarios for these sites being classified as phenotypic ($P$), antigenic ($A$), and pleiotropic ($PA$) sites. Similarly, in our $L=800$ simulations, we let 630 of the sites be nonsynonymous and the remaining 170 sites be synonymous ($S$), again resulting in a nonsynonymous-to-synonymous ratio of 3.7. In all of our simulations, we set the parameter $k$ to 100, such that replicative fitness spans values from 0 to 100 (or a little higher for the most rugged landscapes considered; see Fig. 2E). Unless otherwise noted, we further set infecting genotypes to be $\sim $50% adapted to the host by setting the reference genotype $G^{*}$ to all ones and letting infecting genotypes be bitstrings that contain exactly 50% ones and exactly 50% zeros at randomly chosen sites across their genomes.

We set the mutation rate to $\mu = 2.5 \times 10^{-5}$ mutations per site per infection cycle. This mutation rate lies between the estimated mutation rate of $\sim 10^{-6}$ mutations per site per cycle for coronaviruses (Bar-On et al., 2020, Amicone et al., 2022) and the estimated mutation rate of $\sim 10^{-4}$ mutations per site per cycle for influenza A viruses (Pauly et al., 2017). At this mutation rate and with a genome length of $L = 400$, two or more mutations are expected to occur in less than 0.005% of replications. With a genome length of $L = 800$, two or more mutations are also expected to only rarely occur (in less than 0.02% of replications). As such, in our simulations, we only allow zero mutations (with probability $e^{-\mu L}$) or one mutation (with probability $1-e^{-\mu L}$) to occur during the process of viral replication. We set the generation time to 6 h, corresponding to a death rate of $d = 4$ per day. We chose this generation time based on the 6–8 h generation time estimated for influenza viruses (Einav et al., 2020) and the 6–9 h generation time estimated for SARS-CoV-1 (Schneider et al., 2012, Bar-On et al., 2020). In all of our simulations, we calculate overall fitness values for genotypes using equation (2) and set the time step to $\Delta t = 1$ hour.

## Results

### Viral population size modulates the strength of selection and genetic drift

We first simulated the model to confirm that it recovers the well-established evolutionary pattern that genetic drift dominates when (effective) population sizes are small and that selection acts more efficiently when population sizes are larger. To this end, we simulated viral evolution on a relatively smooth replicative fitness landscape ($c = 0.5$) for two different viral population sizes: $N = 40$ and $N = 5000$. The viral genome in these simulations consisted of 85 synonymous ($S$) sites and 315 phenotypic ($P$) sites. Supplementary Figure S1 shows the evolutionary dynamics of simulated viral populations over the time course of 3 years. When viral population sizes are small ($N = 40$), mean fitness of these populations does not consistently increase (Supplementary Fig. S1A), indicating a lack of viral adaptation. In contrast, when viral population sizes are larger ($N = 5000$), mean population fitness consistently increases (Supplementary Fig. S1B). In small viral populations, genetic divergence accrues at a rate that is similar to that expected under neutral evolution (Supplementary Fig. S1C), whereas in large viral populations, genetic divergence accrues at a rate that exceeds that expected under neutral evolution (Supplementary Fig. S1D), again, indicating the occurrence of viral adaptation in these larger populations. Finally, as one would expect, average pairwise genetic diversity levels are lower in the smaller viral populations (Supplementary Fig. S1E) than in the larger viral populations (Supplementary Fig. S1F). More interestingly, the small populations show patterns of genetic diversity that are consistent with expected levels of genetic diversity under neutral evolution (Supplementary Fig. S1E), whereas the large populations show patterns of genetic diversity that are substantially lower than those expected under neutral evolution (Supplementary Fig. S1F). This is consistent with positive selection acting to reduce genetic diversity in the large-$N$ simulations. Together, the results shown in Supplementary Fig. S1 indicate that viral population sizes modulate the strength of selection and genetic drift, with genetic drift dominating in small viral populations and selection dominating in large viral populations, as expected from population genetic theory.

### Observed excess of nonsynonymous substitutions appears incompatible with viral evolution on static replicative fitness landscapes

We next used our model to explore the impact that fitness landscape ruggedness has on patterns of within-host viral evolution and adaptation, specifically focusing on what types of fitness landscapes could consistently reproduce the empirically observed pattern that nonsynonymous substitution rates generally exceed synonymous substitution rates in prolonged viral infections (Fig. 1A). To this end, we considered four fitness landscapes across a range of ruggedness from $c = 0.03$ (highly rugged) to $c = 0.5$ (smooth), each with an overall viral genome size of $L=400$ sites. In each case, we set the viral population size to $N = 5000$ based on findings that viral effective population sizes in prolonged infections are thought to be large (Xue et al., 2017, Lumby et al., 2020). We further initially considered only synonymous sites and sites impacting replicative fitness ($L_{S} = 85$, $L_{P} = 315$, $L_{A} = 0$, $L_{PA} = 0$) and simulated viral evolution on each of the four fitness landscapes for one-year periods. Six independent replicates were simulated for each fitness landscape to be able to assess general trends.

**Figure 3:**
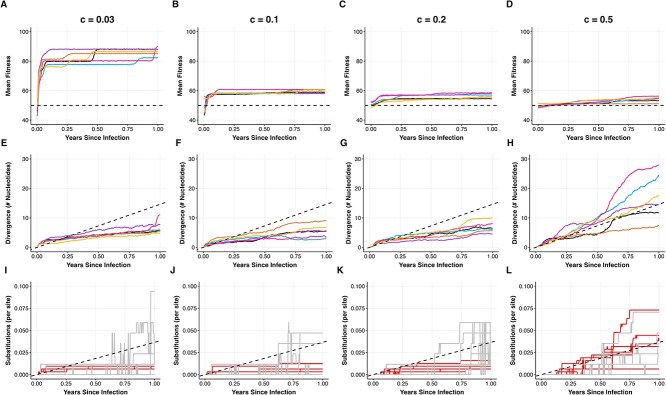
Patterns of viral adaptation observed across fitness landscapes of variable ruggedness. Columns correspond to simulations of viral populations on fitness landscapes of increasing smoothness, with the $c=0.03$ landscape implementing a highly rugged landscape (first column) and the $c=0.5$ landscape (last column) implementing a relatively smooth landscape. (A–D) Mean population fitness over the course of infection for six independent simulations. The dashed black line at 50 indicates the expected fitness of the infecting genotype. (E–H) Mean divergence from the infecting genotype for the same six populations. The dashed black line shows expected divergence under neutral evolution. (I–L) Number of nonsynonymous and synonymous substitutions in the six populations over time. For a given simulation, substitutions are calculated between the consensus sequence at a given time point and the infecting genotype. Red lines show nonsynonymous substitutions. Grey lines show synonymous substitutions. Substitutions are normalized by the number of nonsynonymous and synonymous sites, respectively, yielding a per-site number of substitutions. Dashed black line shows the expected number of substitutions per site under neutral evolution. All simulations used a viral genome of length $L = 400$, with $L_{S} = 85$ and $L_{P} = 315$. Each infecting genotype had a Hamming distance of 200 from the reference genotype of all ones. Other parameters were: $N = 5000$, $\mu = 2.5 \times 10^{-5}$ mutations per site per infection cycle, $k = 100$, $d = 4$ infection cycles per day.

On a highly rugged landscape ($c = 0.03$), viral populations rapidly adapted in the first 2 months following infection, from a mean fitness value of $\sim $50 to a mean fitness value of $\sim $80 (Fig. 3A). Following this initial period of adaptation, further increases in fitness were less pronounced and only occurred sporadically. Divergence from the infecting genotype increased rapidly during the initial period of adaptation but then tended to slow down (Fig. 3E), with divergence considerably lower than expected under neutral evolution during later time points. These results are consistent with initial movements of the viral populations to local higher-fitness peaks in the landscape, followed by the populations getting “trapped” in these local peaks, at least temporarily. Indeed, when we plot changes in these populations’ consensus sequences over time, we see that a small number of nonsynonymous substitutions occurred shortly following infection, but additional nonsynonymous substitutions were rare thereafter (Fig. 3I). In contrast, synonymous substitutions continue to accumulate over the year of simulation (Fig. 3I). Figure 3I further indicates that nonsynonymous viral substitution rates would be expected to be lower than synonymous ones in viral populations that have evolved on this rugged fitness landscape when calculated 6–12 months following infection.

We next simulated viral evolution on a less rugged fitness landscape ($c=0.1$). Mean fitness of the simulated viral populations also rapidly increased within the first 2 months of infection, but fitness levels only reached values of $\sim $60 (Fig. 3B), rather than the fitness values of 80 that were observed on the more rugged fitness landscape of $c=0.03$. This indicates that viral populations still got trapped, at least temporarily, in local fitness peaks on this less rugged landscape, despite the landscape being smoother. The remainder of the evolutionary patterns on the $c=0.1$ landscape are highly similar to those observed on the $c=0.03$ landscape: divergence tended to level off (Fig. 3F) and nonsynonymous substitutions occurred only early on in infection and then purifying selection dominated (Fig. 3J), resulting in fewer nonsynonymous substitutions than synonymous substitutions by 6–12 months post-infection. Patterns of viral evolution on an even smoother fitness landscape ($c=0.2$) look similar to those on the $c=0.1$ landscape, with the only appreciable difference being that the initial periods of viral adaptation that occurred during the first 2 months of infection reached even lower fitness plateaus (55–60, rather than $\sim $60). This pattern of lower fitness plateaus in higher-$c$ simulations makes sense in that local fitness peaks in higher-$c$ simulations will have fitness values similar to those of the infecting genotypes, whereas local fitness peaks in lower-$c$ simulations can have fitness values much higher than those of the infecting genotypes. Finally, on the smoothest fitness landscape considered ($c=0.5$), fitness levels continued to increase over the simulated year-long infections (Fig. 3D). These continuous increases, however, only increased mean fitness slightly. Unlike the populations that evolved on the more rugged landscapes, divergence in the populations evolving on the smooth $c=0.5$ landscape continued to increase, consistent with neutral patterns of divergence (Fig. 3H). Nonsynonymous substitutions also continued to accrue, but only at rates similar to those of synonymous substitutions (Fig. 3L). This is because the fitness impacts of phenotype-impacting mutations on these smooth landscapes were small, approaching nearly-neutral. Nevertheless, selection was still able to act (albeit slowly) on the viral populations evolving on these smooth landscapes, as indicated by the consistent increase in mean fitness in these populations (Fig. 3D). The sustained increase in mean fitness is possible due to the lack of local fitness peaks on this smooth landscape in which the viral populations can temporarily get trapped in. Together, our results indicate that simulations of viral evolution occurring on fitness landscapes of variable ruggedness do not recapitulate patterns of nonsynonymous substitutions being in excess of synonymous substitutions.

### Viral populations evolving on static replicative fitness landscapes do not diversify into substantively distinct lineages during their adaptation

**Figure 4:**
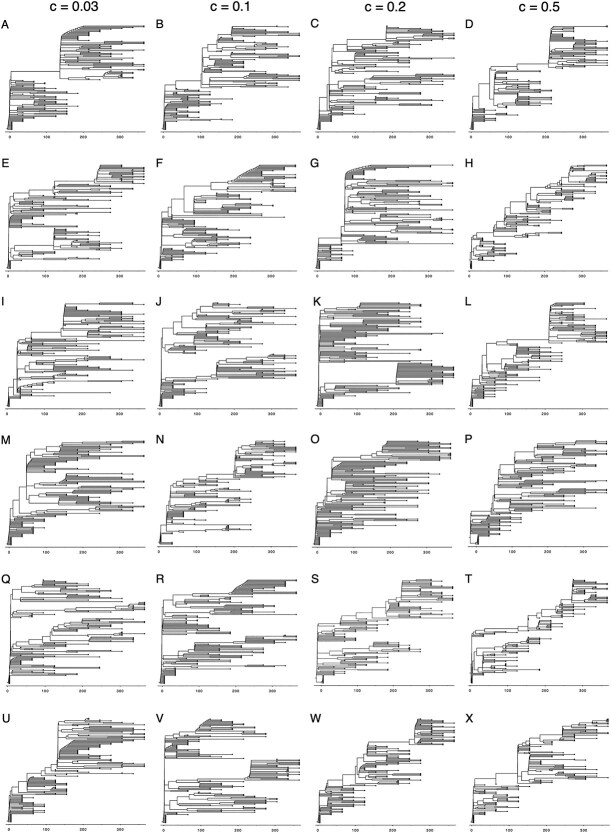
Phylogenies inferred from simulated viral populations evolving on fitness landscapes of variable ruggedness. Columns correspond to the fitness landscapes in Fig. 3, ranging from a highly rugged fitness landscape ($c = 0.03$, first column) to a smooth fitness landscape ($c = 0.5$, fourth column). Rows correspond to the six independent simulations shown in Fig. 3. For each simulation, a time-aligned phylogeny was inferred using a dataset that contained 130 sequences (10 sequences per time point sampled, with monthly sampling from $t=0$ to $t=365$ days). Time-aligned phylogenies were generated by first inferring neighbor-joining trees using the R packages Ape (Paradis and Schliep, 2019) and treeio (Wang et al., 2020) and then using treedater (Volz and Frost 2017) to time-align these neighbor-joining trees. Time-aligned trees were visualized using FigTree v1.4.4 (Rambaut, 2018). The time scale corresponds to the number of days following infection.

While the results shown in Fig. 3 indicate that viral adaptation is expected to occur across fitness landscapes that differ in their ruggedness, they did not yield information on the extent to which the viral populations diversified throughout their adaptation. It is conceivable that the viral populations remained largely monomorphic, in each simulation traversing to a single local fitness peak. Alternatively, viral populations could have diversified, leading to the occupation of many different local fitness peaks. To determine which of these possibilities occurred, we serially sampled the simulated viral populations on a monthly basis and inferred time-aligned trees from these samples (Fig. 4). These time-aligned trees first indicate that viral turnover is generally observed, particularly in viral populations evolving on smoother ($c=0.5$) fitness landscapes. Across all four fitness landscapes considered, the trees do not consistently show viral diversification into substantively distinct lineages. On the more rugged fitness landscapes ($c=0.03$ and $c=0.1$), the most recent common ancestor of viruses sampled at 1-year post infection is sometimes the infecting genotype, but in other simulations, the time of the most recent common ancestor is only several months before this final time point sample. This pattern of recent common ancestry becomes more robust on smoother fitness landscapes (particularly $c=0.5$). As such, these simulations do not consistently reproduce the pattern of viral lineage diversification observed in prolonged infections (Fig. 1B). These phylogenies further indicate that, while several fitness peaks may get “discovered,” the presence of fitness differences between these local peaks often results in viral populations ultimately persisting in only one of them.

### Parallel substitutions do not occur readily on static replicative fitness landscapes

We now address through simulation the question of whether static replicative fitness landscapes of various ruggedness can consistently reproduce the pattern of parallel substitutions that is frequently observed across individuals with prolonged infections (Fig. 1C). We again considered viral evolution on the four different fitness landscapes we considered in Fig. 3, and simulated viral evolution for 6 months. For each landscape, we started each of the six simulations off with the same infecting genotype that was $\sim $50% adapted to the host and used the same fitness landscape for each of the simulations. As such, genotypes that had been accessed in a previous simulation and had their fitness values already dynamically allocated retained their fitness values across simulations.

**Figure 5:**
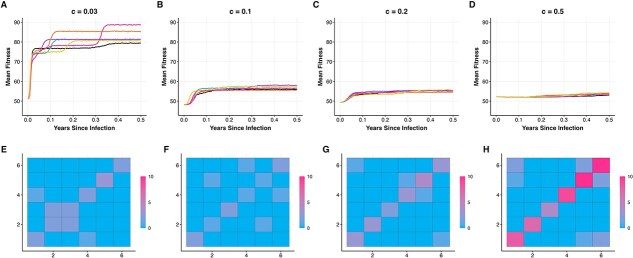
Patterns of viral evolution and parallel mutations on identical static replicative fitness landscapes. In each column, all 6 viral populations evolve on the same static fitness landscape, starting from the same infecting genotype. (A–D) Changes in mean population fitness of six viral populations evolving on fitness landscapes of various ruggedness: $c = 0.03$ (A), $c = 0.1$ (B), $c = 0.2$ (C), and $c = 0.5$ (D). (E–H) The number of shared, high-frequency nonsynonymous mutations observed across pairs of individuals at time $t=0.5$ years. High-frequency was defined as $\geq $20%. Cells along the diagonal show the number of high-frequency nonsynonymous mutations identified in each individual at time $t=0.5$ years. Model parameters are: $N=5000$, $\mu = 2.5 \times 10^{-5}$ mutations per site per infection cycle, $k = 100$, $d = 4$ replications per day, $L_{S} = 85$ and $L_{P} = 315$. In Supplementary Fig. S2, we further considered an alternative definition of what constitutes a high-frequency mutation, defining it as any mutation in an individual that reached a frequency of 20% or higher at any point in time during the 6-month course of the individual’s infection. Parallel mutations were then again considered those that occurred across pairs of individuals. The results using this alternative definition are qualitatively similar to those shown in (E)–(H): few, is any, parallel mutations are observed across pairs of individuals.

On a very rugged landscape ($c=0.03$), the mean fitness of all six simulated viral populations increased, largely in the 2 months following infection (Fig. 5A). These dynamics are, as expected, consistent with the dynamics of mean fitness that were observed across different fitness landscapes parameterized with $c=0.03$ (Fig. 3A). Interestingly, the fitness levels at which the populations plateaued differed across the six simulations, despite the same underlying fitness landscape and the same infecting genotype. This indicates that the different viral populations may, at least temporarily, be residing in different nearby local fitness peaks. To explore this possibility, we identified, for each population, the set of sites that contained a nonsynonymous high-frequency allele that differed from the infecting genotype at 6 months post-infection, defining high-frequency as exceeding 20%. For each pair of individuals, we then determined the number of sites that were shared across their sets. On the $c=0.03$ landscape, we found that very few (if any) high-frequency mutations were shared between individuals (Fig. 5E). To understand these results, we can remember that highly rugged landscapes come close to a House of Cards landscape, where the fitness effect of a mutation depends almost entirely on its genetic context (i.e. epistatic interactions dominate fitness effects). As such, one would expect the first nonsynonymous substitution to impact the fitness effects of all other possible nonsynonymous substitutions. Parallel substitutions would therefore only likely be observed if the first substitution was the same one across individuals. Once different substitutions occurred, the viral populations across the different individuals would be expected to be on different evolutionary paths due to the extreme ruggedness of the fitness landscape.

On a semi-rugged landscape parameterized with $c=0.1$, we see that fitness increases to similar levels across the individual infections (Fig. 5B). However, these fitness increases appear to largely stem from different nonsynonymous substitutions, given little-to-no sharing of nonsynonymous high-frequency mutations (Fig. 5F). Viral evolution on even smoother fitness landscapes again results in little to no sharing of nonsynonymous variation (Fig. 5G and H). These results make sense in that, on smoother landscapes, beneficial mutations all have similar fitness effects, such that there are many different routes to higher fitness.

### Simulated patterns of within-host viral evolution are largely robust to different viral genome sizes

Together, our results shown in Figs 3– 5 indicate that adaptation is readily observed in simulated viral populations evolving on fitness landscapes of variable ruggedness. On rugged landscapes, adaptation occurs rapidly and then evolution is dominated by purifying selection, such that nonsynonymous substitution rates do not exceed synonymous ones in the long-term. On smooth landscapes, adaptation continues to occur, but fitness differentials are so small that nonsynonymous substitution rates are similar to synonymous ones in the long-term. As such, viral evolution on these static fitness landscapes did not consistently reproduce the observed excess of nonsynonymous substitutions observed in prolonged infections. Our simulations on these static landscapes further indicate that co-circulating viral lineages do not robustly evolve and that parallel mutations are not readily observed on any of the considered fitness landscapes. All of these results, however, were based on simulations with a viral genome of size $L=400$ sites. To assess the robustness of these results, we further considered a viral genome with $L=800$ sites. Simulations with this larger genome size were generally consistent with the results with the $L=400$ viral genome. On rough fitness landscapes, $L=800$ viral populations exhibited analogous nonsynonymous and synonymous substitution patterns (Supplementary Fig. S3), an analogous lack of substantive lineage diversification (Supplementary Fig. S4) and an analogous lack of parallel substitutions across individuals experiencing prolonged infection (Supplementary Fig. S5). On smoother fitness landscapes, $L=800$ viral populations again exhibited steady, albeit slow, rates of adaptation (Supplementary Fig. S3). Viral divergence was slightly higher than expected under neutrality, and the rate of nonsynonymous substitutions slightly exceeded that of synonymous substitutions (Supplementary Fig. S3), indicating that this empirical pattern may be reproducible on a smooth fitness landscape if the viral genome size is sufficiently large, provided that a considerable fraction of mutations increase fitness. However, substantive lineage diversification still was not consistently reproduced (Supplementary Fig. S4) and parallel substitutions across individuals experiencing prolonged infection were still not observed (Supplementary Fig. S5) on smooth fitness landscapes with viral genome sizes of $L=800$.

Because of the inability for these viral simulations (with genome sizes of $L=400$ or $L=800$) to consistently replicate observed patterns of within-host viral evolution, we therefore next considered the role that mutations that impact antigenicity may play in the reproduction of the empirical patterns shown in Fig. 1. Specifically, we consider the impact of pleiotropic sites on these patterns, where mutations at these sites impact both replicative fitness and antigenicity.

### Interindividual variation in rates of antigenic evolution can be explained by differences in the strength and breadth of immune pressure

**Figure 6:**
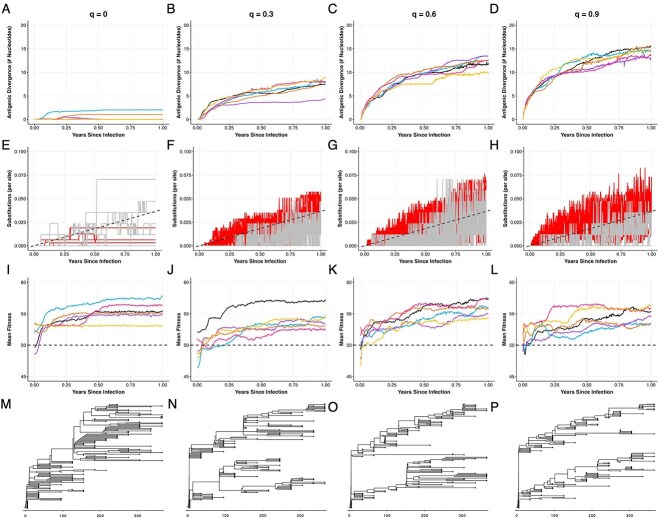
The strength of the immune response impacts patterns of within-host viral evolution in prolonged infections. Columns correspond to varying strengths of the immune response: no immune response ($q = 0.0$), low strength ($q = 0.3$), medium strength ($q= 0.6$), and high strength ($q = 0.9$). All simulations set the breadth of the immune response to $p = 0.8$. (A–D) Extent of antigenic evolution over the course of infection for six simulations. Antigenic evolution was calculated as divergence between the consensus genotype at a given time point and the infecting genotype, at the subset of sites that impact antigenicity ($A$ and $PA$ sites). (E–H) Number of nonsynonymous (red) and synonymous (grey) substitutions per site over the course of infection. Dashed black line shows the expected number of substitutions under neutral evolution. (I–L) Mean viral replicative fitness over the course of infection. The horizontal dashed line shows the expected fitness of the infecting genotype. (M–P) Time-aligned phylogenies for the simulations shown in black in the above panels. The time scale corresponds to the number of days following infection. Simulations were performed using a viral genome of length $L = 400$, with $L_{S} = 85$, $L_{P} = 267$, $L_{PA} = 48$, and $L_{A} = 0$. Other parameters are: $N = 5000$, $\mu = 2.5 \times 10^{-5}$ mutations per site per infection cycle, $k = 100$, $c = 0.2$, and $d = 4$ infection cycles per day.

To assess the impact that pleiotropic sites have on patterns of within-host viral evolution in prolonged infections, we assume a semi-rugged fitness landscape of $c=0.2$, again starting with a viral genome of size $L=400$ with 315 nonsynonymous sites and $L_{S} = 85$ synonymous sites. However, we now assume that a subset of the nonsynonymous sites impact only replicative fitness ($L_{P}=267$) while the remainder of the nonsynonymous sites impact both replicative fitness and antigenicity ($L_{PA}=48$). We arrived at this partition between phenotypic sites and pleiotropic sites based roughly on the proportion of amino acid residues in the receptor binding domain of SARS-CoV-2’s spike gene that impact antigenicity (Greaney et al. 2022). In our simulations, we consider different strengths of immune pressure as well as different breadths of the immune response. Our first simulations consider variation in the strength of immune pressure, modified by varying the value of parameter $q$ in equation (3). Figure 6 shows simulations at four different values of $q$, ranging from $q=0.0$ (no immune pressure) to $q=0.9$ (very strong immune pressure). For all of these simulations we kept immune breadth constant at a moderate level of $p = 0.8$. The strength of immune pressure ($F_{A}$) implemented under these scenarios is shown graphically in Supplementary Fig. S6.

In the absence of immune pressure ($q = 0.0$), antigenic divergence (realized through mutations occurring at pleiotropic sites) remained low (Fig. 6A), as one might expect given the lack of immune pressure acting on these simulated viral populations. In the presence of immune pressure ($q>0$), antigenic divergence increased over the simulated infections, with higher rates of antigenic divergence observed with stronger immune pressure (Fig. 6B-D). In all cases, there is an apparent leveling-off, or plateauing, of antigenic divergence. We return to this intriguing pattern below. While our results show that there is some variation in the amount of antigenic evolution that occurs within simulations that are parameterized with the same strength of immune pressure, there is considerably more variation in the amount of antigenic evolution observed across simulations that differ in the strength of immune pressure. Observed interindividual heterogeneity in the rate of antigenic evolution could thus be explained by variation across individuals in the strength of their immune response, with individuals that exert higher immune pressure giving rise to viral populations that have undergone more antigenic evolution. Of note, these simulations do not consider the impact of immune pressure on viral population sizes; with higher immune pressure, it could be the case that viral population sizes are reduced, which would decrease the efficiency of selection and therefore may reduce the rate of within-host antigenic evolution, as has been suggested previously (Grenfell et al., 2004).

**Figure 7:**
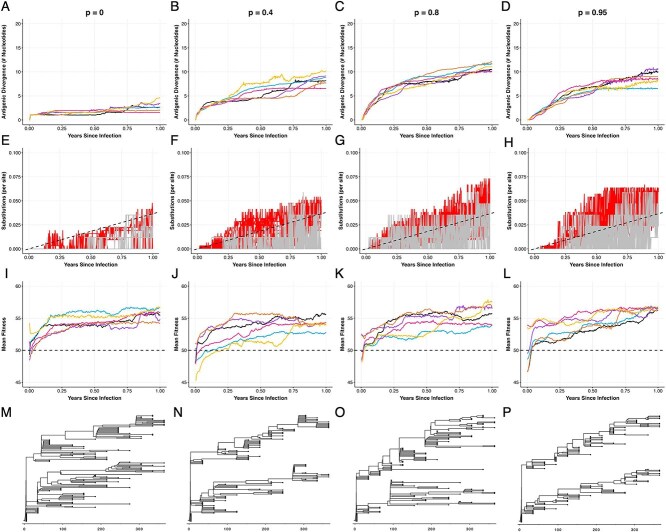
The breadth of the immune response impacts patterns of within-host viral evolution in prolonged infections. Columns correspond to varying breadths of the immune response: very narrow breadth ($p = 0.0$), low breadth ($p = 0.4$), medium breadth ($p = 0.8$), and broad breadth ($p = 0.95$). All simulations assumed a moderate strength of immune pressure ($q = 0.5$). (A–D) Extent of antigenic evolution over the course of infection for six simulations. (E–H) Number of nonsynonymous (red) and synonymous (grey) substitutions per site over the course of infection. Dashed black line shows the expected number of substitutions under neutral evolution. (I–L) Mean viral replicative fitness over the course of infection. The horizontal dashed line shows the expected fitness of the infecting genotype. (M–P) Time-aligned phylogenies for the simulations shown in black in the above panels. The time scale corresponds to the number of days. Simulations were performed using a viral genome of length $L = 400$, with $L_{S} = 85$, $L_{P} = 267$, $L_{PA} = 48$, and $L_{A} = 0$. Other parameters are: $N = 5000$, $\mu = 2.5 \times 10^{-5}$ mutations per site per infection cycle, $k = 100$, $c = 0.2$, and $d = 4$ infection cycles per day.

Figure 7 considers the impact that the breadth of the immune response has on the rate of antigenic evolution. We parameterize the simulations for this figure using different values of $p$ (see equation (3)), ranging from $p = 0.0$ (very narrow immune breadth) to $p = 0.95$ (broad immune breadth). In these simulations, we keep the strength of immune pressure the same across simulations at a moderate value of $q=0.5$. The extent of immune pressure ($F_{A}$) adopted under these scenarios is shown graphically in Supplementary Fig. S7. In simulations with narrow immune breadth ($p = 0.0$), antigenic evolution occurred, but only at a very slow rate (Fig. 7A). As immune breadth increased (higher $p$), the rate of antigenic evolution increased (Fig. 7B and C). These results make sense in that viral genotypes that are more than a single antigenic mutation away from the consensus genotype have incrementally higher antigenic fitness ($F_{A}$) when immune breadth is intermediate, thereby facilitating antigenic divergence. As immune breadth increases further (toward $p = 1.0$), the fitness differential conferred by antigenicity-impacting mutations is reduced. Interestingly, we therefore see a reduced rate of antigenic evolution at very broad immune breadths (Fig. 7D). In Fig. 7C and D, there is again an apparent leveling-off, or plateauing, of antigenic divergence, which we return to below. Our results indicate that observed interindividual heterogeneity in the rate of antigenic evolution could be explained by variation across individuals in the breadth of their immune response, with individuals that have an intermediate immune breadth expected to give rise to viral populations that have undergone the most antigenic evolution. Again, these conclusions are based on results that assume that viral population sizes are not impacted by the breadth of the immune response.

In sum, our results shown in Figs 6A–D and 7A–D indicate that differences between individuals in the strength and/or breadth of their immune response can reproduce heterogeneities in the extent of antigenic evolution that are observed across individuals experiencing prolonged infections (Fig. 1D). To determine whether these results are robust to changes in viral genome size, we again simulated viral evolution under the same immune escape parameterizations, this time with a viral genome that had $L=800$ sites, keeping the same proportions of phenotypic, pleiotropic, antigenic, and synonymous sites as for our previous simulations with $L=400$ sites. Patterns of antigenic evolution in these simulations displayed the same overall trends as those observed in Figs 6 and 7, with more antigenic evolution observed when the strength of the immune response was stronger (Supplementary Fig. S8) and the highest amount of antigenic evolution observed when the breadth of the immune response was moderate (Supplementary Fig. S9). However, because of the larger number of sites that impacted antigenicity in the $L=800$ simulations, the overall levels of antigenic divergence tended to be larger in the $L=800$ simulations than in the $L=400$ simulations.

We next wanted to determine whether and to what extent the simulated patterns of antigenic evolution would be robust to whether the antigenicity-impacting mutations affected only antigenicity or whether they affected both antigenicity and replicative fitness (i.e. whether the antigenicity-impacting sites were antigenic ($A$) sites or pleiotropic ($PA$) sites). To address this question, we simulated the model with our original $L=400$ site viral genomes, under the same parameterizations as those in Figs 6 and 7, with the only difference being that the $L_{PA} = 48$ pleiotropic sites were replaced with $L_{A} = 48$ antigenic sites (Supplementary Figs S10 and S11). Simulations of this model yielded similar patterns of antigenic evolution to those shown in Figs 6 and 7, with the greatest amount of antigenic evolution occurring when immune pressure was strong (high $q$, Supplementary Fig. S10A–D) and when immune breadth was intermediate (intermediate $p$, Supplementary Fig. S11A–D). A closer comparison of Supplementary Fig. S10 and Fig. 6, however, reveals that when the antigenicity-impacting sites also impact viral replicative fitness (i.e. when $L_{PA}=48$ and $L_{A}=0$; Fig. 6A–D), the rate of antigenic evolution is slower than when antigenicity-impacting sites only impact antigenicity (i.e. when $L_{PA}=0$ and $L_{A}=48$; Supplementary Fig. S10A–D). This is likely because antigenic evolution is impeded by the simultaneous impact these mutations have on viral replicative fitness. Similarly, a closer comparison of Supplementary Fig. S11 and Fig. 7 reveals that when the antigenicity-impacting sites also impact viral replicative fitness (i.e. when $L_{PA}=48$ and $L_{A}=0$; Fig. 7A–D), the rate of antigenic evolution is slower than when antigenicity-impacting sites only impact antigenicity (i.e. when $L_{PA}=0$ and $L_{A}=48$; Supplementary Fig. S11A–D). These results, at the within-host level, are consistent with findings from a recent study that showed that pleiotropic effects can constrain the antigenic evolution of influenza viruses at the population level (Yu et al., 2025).

### Immune pressure increases nonsynonymous substitution rates

We now revisit the patterns of viral evolution shown in Fig. 1A–C to assess whether our above simulations with antigenicity-impacting sites can consistently reproduce these patterns. We do this by returning to the $L=400$ simulations, with $L_{P} = 267$, $L_{PA}=48$, $L_{A}=0$, and $L_{S} = 85$. Figure 6E–H indicate that when the strength of the immune response is greater (larger $q$), nonsynonymous substitution rates increase and exceed synonymous substitution rates. This result makes sense in that mutations at pleiotropic sites can and often do yield viral genotypes that have a selective advantage in the presence of immune pressure, with the selective advantage being larger with stronger immune pressure. Figure 7E–H indicate that when immune breadth is at an intermediate level (moderate $p$), nonsynonymous substitution rates are higher than they are at either narrow breadth or broad breadth, and again exceed synonymous substitution rates. This result similarly makes sense in that mutations at pleiotropic sites again yield viral genotypes that experience the largest selective advantage when immune breadth is at an intermediate level.

While immune escape, occurring through mutations at pleiotropic sites, consistently increases nonsynonymous substitution rates, its impact on viral replicative fitness is varied. When infecting genotypes are of intermediate fitness ($\sim $50% adapted), mean replicative fitness increases similarly across simulations without immune pressure (Fig. 6I) as with immune pressure of various strengths and breadths (Figs 6J–L and 7I–K, 7L). Under these scenarios, therefore, immune escape neither impedes nor facilitates viral phenotypic adaptation. The presence of host immune pressure, however, does impact viral replicative fitness when the infecting genotype is either poorly adapted to the host or when it is well-adapted to the host. When the infecting genotype is poorly adapted to the host, stronger immune pressure tends to facilitate viral phenotypic adaptation, with mean fitness levels increasing to higher levels than in the absence of immune pressure (Supplementary Fig. S12). In contrast, when the infecting genotype is well adapted to the host, stronger immune pressure tends to impede viral phenotypic adaptation, with mean fitness levels evolving to be lower than in the absence of immune pressure (Supplementary Fig. S13). These results make sense in terms of immune pressure driving antigenic evolution. When antigenic evolution occurs at pleiotropic sites, then these evolutionary dynamics increase mean replicative fitness if the replicative fitness effect of these mutations are on average positive, which they are if the infecting genotype is poorly adapted. In contrast, these evolutionary dynamics decrease mean replicative fitness if the replicative fitness effect of these mutations are on average negative, which they are if the infecting genotype is well adapted. As such, we would not expect immune pressure to either always facilitate or impede viral adaptation unrelated to antigenicity. The net impact of immune pressure on viral adaptation unrelated to antigenicity is expected to depend on the average replicative fitness impact of mutations at pleiotropic sites.

### Immune pressure results in multiple co-circulating viral lineages

In the absence of sites impacting antigenicity, our simulations did not robustly reproduce empirically observed patterns of co-circulating viral lineages. However, in the presence of immune pressure, our simulations consistently yielded two, and occasionally three, co-circulating viral lineages (Figs 6N–P and 7M–P). This was the case for even the lowest extent of immune strength we considered ($q=0.3$; Fig. 6N) and for even relatively broad immunity breadth ($p = 0.95$; Fig. 7P). These results make sense in that immune pressure, as implemented, results in negative frequency-dependent selection. As such, the consensus genotype is expected to shift back and forth between the two clades, with the viral clade that does not include the consensus genotype having a selective advantage.

The co-circulation of viral lineages that are observed in the presence of immune pressure also help us understand the seemingly jagged nonsynonymous and synonymous substitution rates apparent in Figs 6F–H and 7E–H. These occur because of co-circulating clades that have different numbers of nonsynonymous as well as synonymous substitutions. The jaggedness appears from the consensus genotype rapidly changing from being in one clade to being in another clade.

Finally, the co-circulation of viral lineages in these simulations also help us understand the plateauing of antigenic divergence that is observed in Figs 6B–D and 7B–D. This plateauing results from the diminishing returns of immune escape mutations combined with negative frequency dependent selection. More specifically, if an immune escape mutation occurs in a viral genome that is in the non-dominant clade (i.e. in the clade that does not contain the consensus sequence), then this mutation would not appreciably increase antigenic fitness ($F_{A}$) because both the mutant virus and its parent would both already be antigenically far away from the consensus genotype. If an immune escape mutation occurs in a viral genome that is instead in the dominant clade, then this mutation would increase its antigenic fitness, but only while that clade remains dominant. As such, once two (or more) antigenically divergent lineages are co-circulating, immune pressure for new antigenic mutations should be reduced, resulting in a leveling-off of antigenic divergence. Note that this expectation may be specific to the way we model antigenic fitness; if we did not model immune escape as “fleeting” (see Methods), but instead assumed that viral antigenic fitness depended in some way on the history of viral genotypes that have circulated over the course of infection, we might not expect antigenic divergence to plateau.

**Figure 8:**
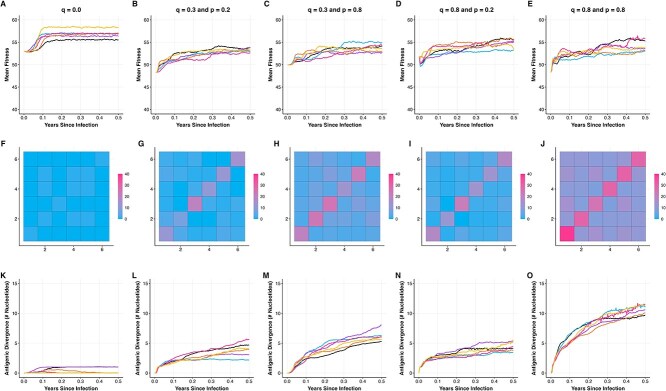
Immune pressure increases the frequency of parallel mutations observed across individuals. Columns correspond to different parameterizations of the immune response (depicted in Fig. 1H). Column 1: no immune pressure ($q=0.0$). Column 2: weak immune strength ($q=0.3$) and narrow immune breadth ($p = 0.2$). Column 3: weak immune strength ($q=0.3$) and moderate immune breadth ($p = 0.8$). Column 4: strong immune strength ($q=0.8$) and narrow immune breadth ($p = 0.2$). Column 5: strong immune strength ($q=0.8$) and moderate immune breadth ($p = 0.8$). Infecting genotypes are $\sim $50% adapted to the host. (A–E) Changes in mean viral replicative fitness for six independent viral populations evolving on the same fitness landscape, starting with the same infecting genotype. (F–J) The number of shared high-frequency mutations across pairs of individuals. Only mutations at nonsynonymous sites that exceeded frequencies of 20% at time $t=0.5$ years were considered in this calculation. Supplementary Figure S14 shows analogous results for an alternative definition of high-frequency nonsynonymous mutations, namely any nonsynonymous mutation that reaches a frequency of 20% or higher at any point over the 6-month course of an individual’s infection. (K–O) Extent of antigenic evolution over the course of infection. All simulations were performed using viral genome of length $L = 400$, with $L_{S} = 85$, $L_{P} = 267$, $L_{PA} = 48$, and $L_{A} = 0$. Other parameters are: $N = 5000$, $\mu = 2.5 \times 10^{-5}$ mutations per site per infection cycle, $k = 100$, $c = 0.2$, and $d = 4$ infection cycles per day.

### Immune pressure increases the likelihood of observing parallel substitutions

Finally, we examined the impact of immune pressure on the occurrence of parallel mutations across individuals. To compare against simulations that included immune pressure, we first simulated a model with no immune pressure by setting the strength of immune pressure to $q=0.0$. Consistent with our previous results, mean replicative fitness in each individual increased over the 6 months of simulation (Fig. 8A). Next, we calculated the number of parallel high-frequency mutations that were shared across individuals at time $t=0.5$ years, as we did for Fig. 5. We again considered only nonsynonymous sites and defined high-frequency mutations as those that exceeded 20%. Figure 8F again shows that in the absence of immune pressure ($q=0.0$), parallel mutations do not readily occur. (The results shown in Fig. 8F are quantitatively similar to those in Fig. 5G, but the scale bar used is different, such that the results in Fig. 8F can be compared against those of Fig. 8G–J.) In contrast, in the presence of immune pressure, parallel mutations are more readily observable (Fig. 8G–J). This is particularly the case when the strength of immune pressure is strong and when the breadth of the immune response is moderate (Fig. 8J). This is because the rate of antigenic evolution is largest under this parameterization (Fig. 8O versus Fig. 8K–N). We hypothesized that the reason why parallel mutations were more likely to be observed in the presence of immune pressure was because there were relatively few sites that impacted antigenicity and even fewer of these sites that impacted antigenicity and did not decrease replicative fitness. To test this hypothesis, we simulated the model under a similar parameterization, only changing the 48 pleiotropic ($PA$) sites into antigenic ($A$) sites. Supplementary Fig. S15 shows that simulations of this model generate similar patterns to those shown in Fig. 8F–J, with somewhat higher frequencies of observed parallel mutations in the simulations with antigenic $A$ sites rather than pleiotropic ($PA$) sites. The greater number of parallel mutations in Supplementary Fig. S15 is because the overall rate of antigenic change is higher when the evolution of antigenicity-impacting mutations is not constrained by pleiotropic effects. As such, pleiotropic effects tend to *decrease* the number of observed parallel mutations due to the constraints they place on the rate of antigenic evolution. To further determine whether parallel mutations occur as a result of a small number of antigenicity-impacting sites, we next performed model simulations with a much larger number of antigenicity-impacting mutations, while keeping the viral genome size at $L=400$ sites: $L_{P} = 48$, $L_{PA} = 267$, $L_{A} = 0$, and $L_{S} = 85$. Supplementary Figure S16 shows that simulations of this model, even in the presence of strong immune pressure, do not result in a large number of parallel mutations. This indicates that, indeed, the common occurrence of parallel mutations observed in Fig. 8 in the presence of immune pressure is due to the relative small number of sites that impact antigenicity and the strength of selection for antigenic change in these simulations.

## Discussion

Prolonged infections with respiratory viruses such as influenza viruses and coronaviruses have been extensively documented. Through serial sampling of these infections, several consistent patterns of viral evolution have been identified, including an observed excess of nonsynonymous substitutions (Fig. 1A), co-circulating viral lineages (Fig. 1B), parallel substitutions across infected individuals (Fig. 1C), and variable rates of antigenic evolution (Fig. 1D). Here, we have developed a fitness landscape model to determine what processes likely drive these evolutionary patterns. Through simulation, we found that the patterns shown in Fig. 1A–C could not be consistently reproduced in the absence of sites that impacted antigenicity. In contrast, when we instead assumed that a subset of the nonsynonymous sites impacted antigenicity in addition to viral replicative fitness, our simulations were able to consistently reproduce these three observed patterns. The final pattern, of variable rates of antigenic evolution, could be explained by either differing strengths of immune pressure across infected individuals and/or differing breadths of the immune response across infected individuals.

We designed our model to be flexibly parameterized by easily changing the number and types of sites, the mutation rate, the ruggedness of the replicative fitness landscape, and the strength and breadth of the immune response. While we could not simulate the model under all possible parameterizations, we considered the impact of multiple different fitness landscapes on patterns of within-host viral evolution, the impact of different viral genome sizes and the impact of different strengths and breadths of the immune response on these patterns. Our model did adopt several assumptions that could be relaxed in future studies. First, we assumed that no recombination occurs within our model genome. This assumption simplified our model considerably, in that we did not have to model cellular co-infection either implicitly or explicitly, and it allowed us to organize our sites in the model genome in an arbitrary order. While this assumption simplified our model, it also prevented us from being able to determine how different fitness landscapes and types of immune pressure would impact rates of recombination and ultimately adaptation and immune escape. Second, we assumed that viral population sizes were constant over the course of infections and compared across simulations that had the same viral population size. This assumption could be easily relaxed, but in the absence of a question related specifically to population sizes and population dynamics, we adopted this assumption to be able to interpret our results without this variation as a confounding factor. Third, we assumed a single viral population within each individual, with no spatial subdivision or tissue compartmentalization. As such, spatial structure could not be assessed or invoked as a driver of any of the evolutionary patterns we considered. Given findings that within-host spatial structure occurs in respiratory virus infections and may contribute to genetic diversification of within-host viral populations (Gallagher et al., 2018, Chaguza et al., 2023, Farjo et al., 2024, Smith et al., 2024, Ferreri et al., 2025), this assumption could be relaxed in future extensions of the model. Third, we assumed that all mutations that impacted antigenicity did so to the same extent. This is clearly not the case empirically. For example, for influenza viruses, it is well known that mutations around the receptor binding site have particularly large antigenic effects, although mutations at other sites in the head of the hemagglutinin protein also impact antigenicity (Koel et al., 2013). This variation in antigenic impact may explain why, of the parallel mutations observed across individuals experiencing prolonged infections, there are several ones (like SARS-CoV-2’s E484K mutation) that are particularly recurrent. Finally, we used our model to evaluate the drivers of four different evolutionary patterns that have been documented in prolonged infections with respiratory viruses. As additional studies accrue, some of these patterns themselves may need reevaluation. For example, a recent study has found that sequencing errors may have inflated estimates of viral diversity and evolutionary rates of SARS-CoV-2 populations sampled from individuals experiencing prolonged infections (Rutsinsky et al., 2024).

Although the work presented here focused on viral evolution in individuals experiencing prolonged infection with respiratory viruses that generally cause acute infection, our results reveal patterns similar to those observed in other prolonged viral infections, including prolonged infections with enteric viruses such as noroviruses (Beek et al., 2017, Doerflinger et al., 2017) as well as with viruses such as hepatitis C virus (HCV) and HIV that lead to chronic infection. For example, chronic HCV infections are known to result in rapid diversification from the founder virus into multiple distinct viral lineages that continue to cocirculate over the course of the infection (Raghwani et al., 2016  2019). This co-circulation of viral lineages results in significant fluctuations in viral divergence over time (Raghwani et al., 2016), a pattern that we also observed in our analysis when we incorporated immune pressure. As such, we hope that the fitness landscape model presented here may further be adapted to evaluate the drivers of observed patterns of viral evolution in these other types of viruses.

## Supplementary Material

rough_MountFufi_final_supplemental_clean_veaf054

## Data Availability

All simulation code is available on GitHub at: https://github.com/katiakoelle/rmf_model. All data in this work are simulated data.
