# Supplementary material for: Immune pressure is key to understanding observed patterns of respiratory virus evolution in prolonged infections
Source: Virus Evol. 2025 Jul 21;11(1):veaf054. doi: 10.1093/ve/veaf054 (PMC12360705; doi:10.1093/ve/veaf054)
Supplement: rough_MountFufi_final_supplemental_clean_veaf054 [file rough_mountfufi_final_supplemental_clean_veaf054.pdf]

Immune Pressure is Key to Understanding Observed Patterns of Respiratory Virus Evolution in Prolonged Infections

Supplemental Material

Amber Coats[1,*], Yintong Rita Wang[2], Katia Koelle[2,3,*]

1 Program in Microbiology and Molecular Genetics, Emory University, Atlanta, GA

2 Department of Biology, Emory University, Atlanta, GA

3 Emory Center of Excellence for Influenza Research and Response (CEIRR), Atlanta GA, USA

* amber.nicole.coats@emory.edu, katia.koelle@emory.edu

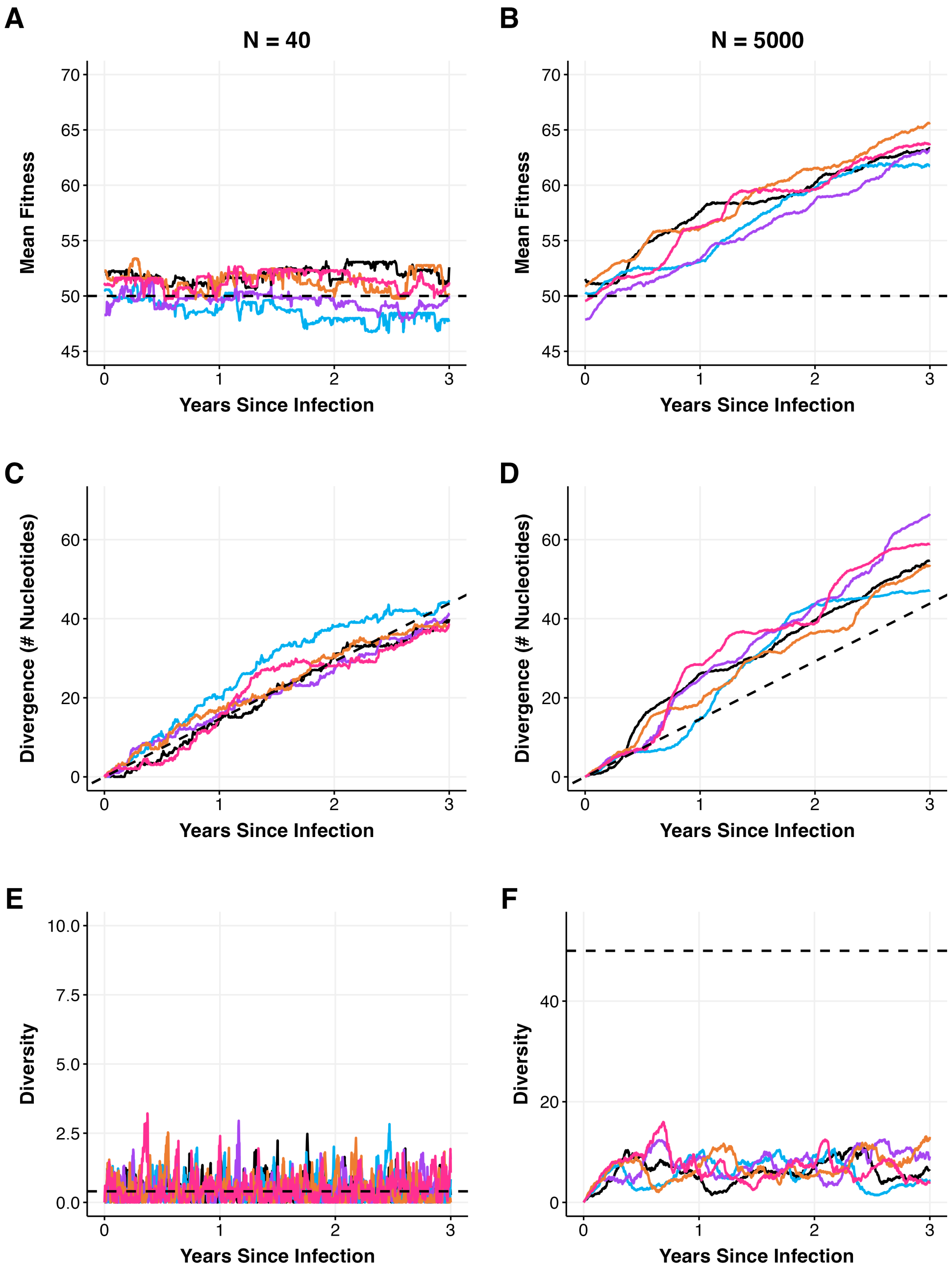

Figure S1. Genetic drift and selection in smaller versus larger within-host viral populations. The first column shows viral evolutionary dynamics of five independently simulated prolonged infections where the viral population size is $N = 40$. The second column shows viral evolutionary dynamics of five independently simulated prolonged infections where the viral population size is $N = 5000$. (A,B) Mean replicative fitness of evolving viral populations. Dashed black lines show expected fitness of the infecting genotype. (C,D) Mean nucleotide divergence of the populations shown in (A,B). Dashed black lines show expected divergence under neutral evolution, given by $\mu L t$, where L is the genome length, time t is measured in years, and the mutation

rate, converted to the time scale of years, is: $\mu = 0.0365$ mutations per site per year ($= 2.5 \times 10^{-5}$ mutations per site per replication cycle $\times$ 4 replication cycles per day $\times$ 365.25 days per year). (E,F) Mean pairwise diversity of the populations shown in (A,B). Mean pairwise diversity is calculated as: $\sum_i \sum_j [x_i x_j D(G_i, G_j)]$, where x_i is the frequency of genotype i, x_j is the frequency of genotype j, and $D(G_i, G_j)$ is the Hamming distance between genotypes i and j. This is equivalent to multiplying the per-site average genetic diversity π (classically given in equation [22] in Nei and Li (1979)) by the total number of sites L. The dashed black lines show expected pairwise diversity levels under neutral evolution at equilibrium. Expected pairwise diversity (of a haploid population evolving under a Moran model at equilibrium) is given by: $\mu \times L \times N$. The evolutionary dynamics of all viral populations were simulated for three years. All simulations used a genome length of $L = 400$ nucleotides, with synonymous $L_S = 85$ sites and phenotypic $L_P = 315$ sites ($L_A = 0$, $L_{PA} = 0$). Other parameters are: $c = 0.5$, $\mu = 2.5 \times 10^{-5}$ mutations per site per replication cycle, $k = 100$, $d = 4$ per day, and $\Delta t = 1$ hr. Each infecting genotype had a Hamming distance of exactly 200 nucleotides from the reference genotype G^* of all ones. The sites of the 200 one alleles and the 200 zero alleles were randomly chosen for each infecting genotype. Note that we present these $N = 40$ and $N = 5000$ simulations only to illustrate how different viral population sizes impact the relative importance of genetic drift and selection in contributing to intrahost viral evolutionary dynamics. Although intrahost viral effective population sizes in acute respiratory viral infections are also thought to be small (on the order of tens to hundreds), we do not consider the $N = 40$ simulations here to reflect patterns that we may expect in these acute infections. This is because the time frame of acute infections is much shorter and because viral populations rapidly expand (by many orders of magnitude) and decline (by similar orders of magnitude) over the time course of days in these acute infections and all of our simulations assume a constant viral population size.

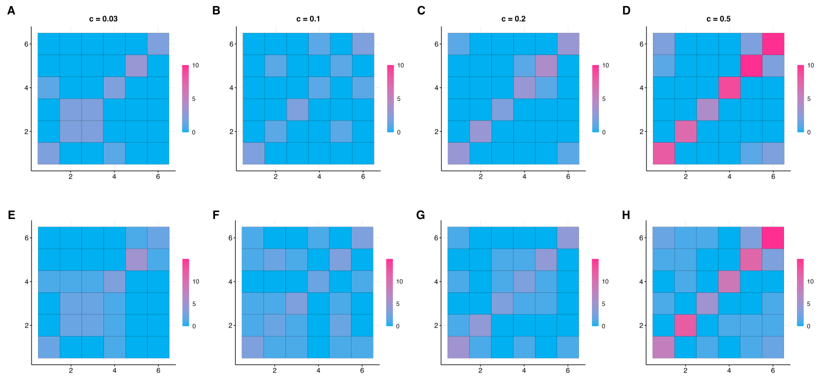

Figure S2. Patterns of parallel mutations under different definitions of what constitutes a high-frequency mutation. (A)-(D) Number of shared, high-frequency nonsynonymous mutations observed across pairs of individuals at time $t = 0.5$ years, reproduced from Figure 5 (panels E-H), for viral populations evolving on fitness landscapes of ruggedness $c = 0.03$, $c = 0.1$, $c = 0.2$, and $c = 0.5$, respectively. Only mutations that exceeded 20% at time $t = 0.5$ years were considered high-frequency. (E)-(H) Number of shared, high-frequency nonsynonymous mutations observed across pairs of individuals using an alternative approach for identifying the set of high-frequency nonsynonymous mutations in each individual. Here, mutations were considered high-frequency if they reached frequencies of $\geq$20% at any point in time over the course of infection. The number of identified high-frequency mutations in each individual is, as expected, higher when all mutations that exceeded 20% frequency at any point over the course of infection are considered, rather than only those that exceeded 20% frequency at $t = 0.5$ years following infection. This pattern can be seen by the higher numbers along the diagonal in panels (E)-(H) compared to those in panels (A)-(D). However, results are similar along the off-diagonals, indicating that parallel mutations occur rarely, if at all, across pairs of individuals, regardless of the definition of what constitutes a high-frequency mutation.

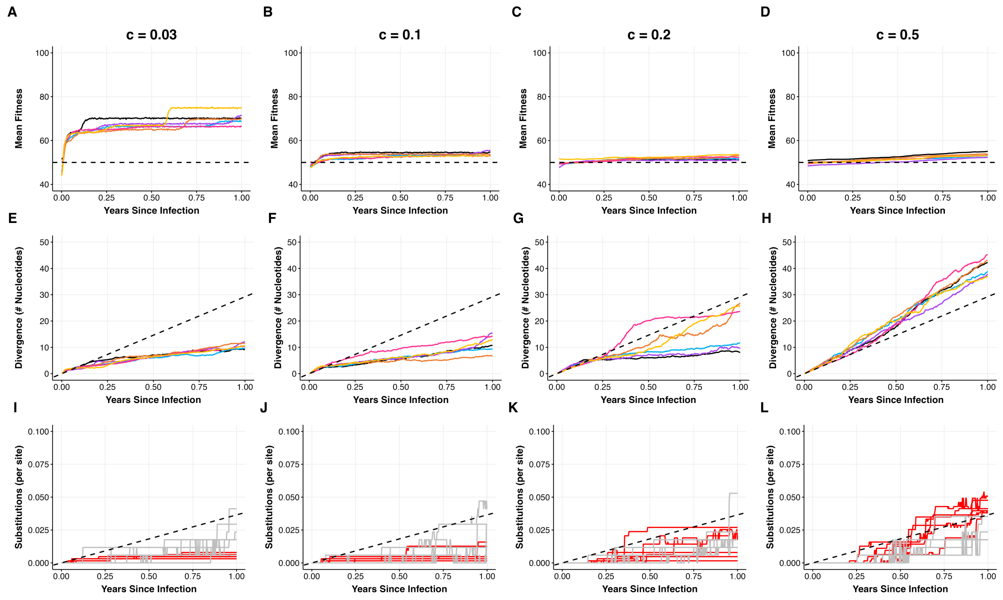

Figure S3. Patterns of viral adaptation observed across fitness landscapes of variable ruggedness. Here, the viral genome size is $L = 800$, with $L_P = 630$, $L_{PA} = 0$, $L_A = 0$, and $L_S = 170$. Columns correspond to simulations of viral populations on fitness landscapes of increasing smoothness, with the $c = 0.03$ landscape implementing a highly rugged landscape (first column) and the $c = 0.5$ landscape (last column) implementing a relatively smooth landscape. (A-D) Mean population fitness over the course of infection for six independent simulations. The dashed black line at 50 indicates the expected fitness of the infecting genotype. (E-H) Mean divergence from the infecting genotype for the same six populations. The dashed black line shows expected divergence under neutral evolution. (I-L) Number of nonsynonymous and synonymous substitutions in the six populations over time. For a given simulation, substitutions are calculated between the consensus sequence at a given time point and the infecting genotype. Red lines show nonsynonymous substitutions. Grey lines show synonymous substitutions. Substitutions are normalized by the number of nonsynonymous and synonymous sites, respectively, yielding a per-site number of substitutions. Dashed black line shows the expected number of substitutions under neutral evolution. Each infecting genotype had a Hamming distance of 400 from the reference genotype of all ones. Other parameters were: $N = 5000$, $\mu = 2.5 \times 10^{-5}$ mutations per site per infection cycle, $k = 100$, $d = 4$ infection cycles per day. Similar to our results from Figure 3, we here observe initially rapid viral adaptation followed by plateauing of viral fitness on highly rugged landscapes ($c = 0.03$) (panel A). Mean population fitness plateaus at a lower level ($\sim$70) when $L = 800$ compared to when $L = 400$ (where mean population fitness plateaus at a value of $\sim$85). This difference may arise because of the higher dimensionality of the Rough Mount Fuji landscape when $L = 800$. At higher dimensionality, there is more opportunity to get trapped (at least temporarily) in local fitness peaks. With $L = 800$, viral divergence on rugged landscapes again levels off (panel E), analogous to what was observed with $L = 400$ (Figure 3E). This leveling off of divergence again stems from nonsynonymous substitutions no longer accruing in the viral population, although synonymous substitutions continue to accrue. As a result, on rugged landscapes, the nonsynonymous substitution rate again does not exceed the synonymous substitution rate. On the smoothest landscape considered ($c = 0.5$), we here again observe steady, albeit slow, increases in viral population fitness over time (panel D), similar to what we

observed when the genome size was $L = 400$ sites (Figures 3D). Divergence continued to increase on this smoother landscape (panel H), again consistent with our $L = 400$ results (Figure 3H) and reflecting the occurrence of weak positive selection. Nonsynonymous substitution rates were slightly elevated above synonymous substitution rates on this landscape panel L), indicating that a larger viral genome ($L = 800$ versus $L = 400$) may allow for positive selection to act more efficiently, given a larger number of sites that could increase viral fitness.

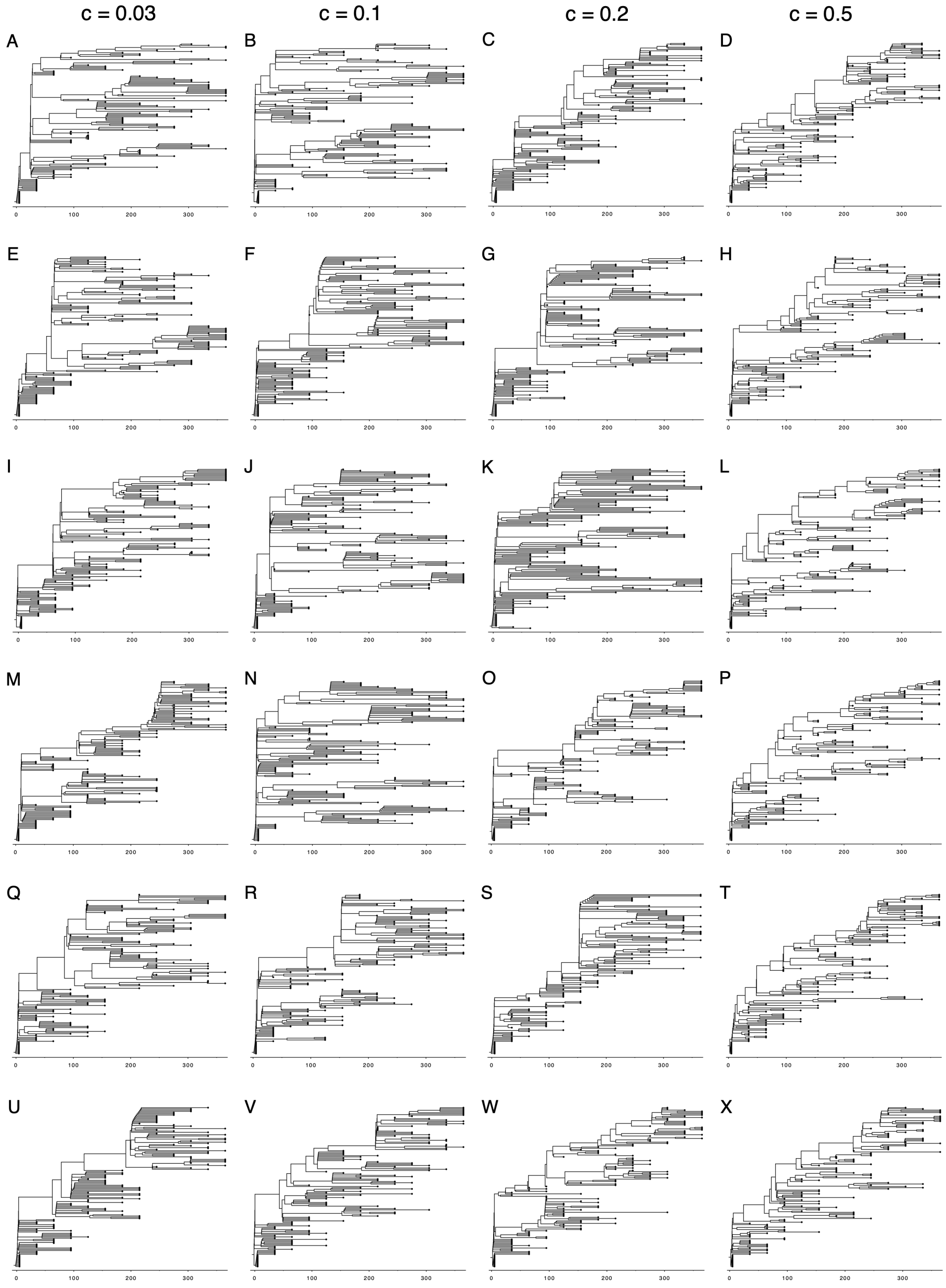

Figure S4. Phylogenies inferred from simulated viral populations evolving on fitness landscapes of variable ruggedness. Here, the viral genome size is $L = 800$, partitioned into $L_P = 630$, $L_{PA} = 0$, $L_A = 0$, and $L_S = 170$. Columns correspond to the fitness landscapes in Figure S3, ranging from a highly rugged fitness landscape ($c = 0.03$, first column) to a smooth fitness landscape ($c = 0.5$, fourth column). Rows correspond to the six independent simulations shown in Figure S3. For each simulation, a time-aligned phylogeny was inferred using a dataset that contained 130 sequences (10 sequences per time point sampled, with monthly sampling from $t = 0$ to

$t = 365$ days). Inferred phylogenies for these $L = 800$ simulations appear topologically similar to those inferred for the $L = 400$ simulations, with a lack of substantive lineage diversification.

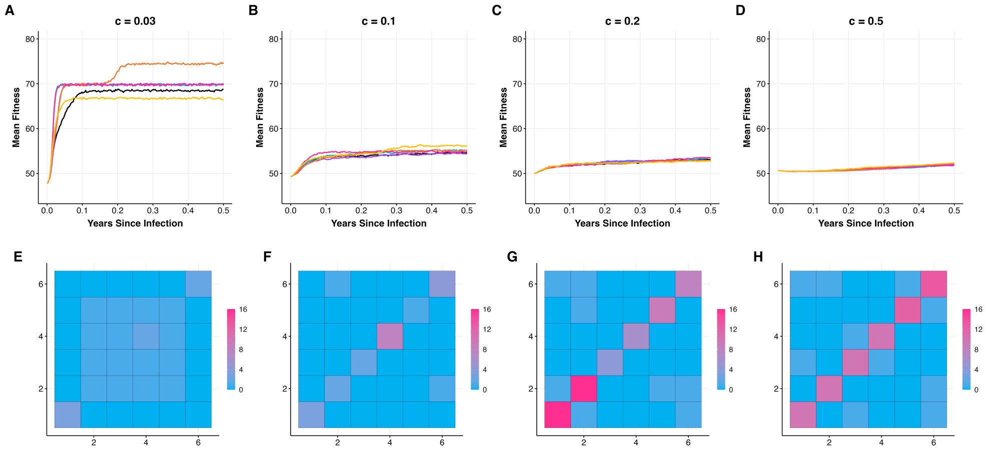

Figure S5. Patterns of viral evolution and parallel mutations on identical static replicative fitness landscapes. Here, the viral genome size is $L = 800$, partitioned into $L_P = 630$, $L_{PA} = 0$, $L_A = 0$, and $L_S = 170$. In each column, all 6 viral populations evolve on the same static fitness landscape, starting from the same infecting genotype. (A-D) Changes in mean population fitness of six viral populations evolving on fitness landscapes of various ruggedness: $c = 0.03$ (A), $c = 0.1$ (B), $c = 0.2$ (C), and $c = 0.5$ (D). (E-H) The number of shared, high-frequency nonsynonymous mutations observed across pairs of individuals at time $t = 0.5$ years. High-frequency was defined as $\geq$20%. Cells along the diagonal show the number of high-frequency nonsynonymous mutations identified in each individual at time $t = 0.5$ years. Model parameters are: $N = 5000$, $\mu = 2.5 \times 10^{-5}$ mutations per site per infection cycle, $k = 100$, and $d = 4$ replications per day. Results from these $L = 800$ simulations are similar to those from the $L = 400$ simulations (Figure 5 in main text), again indicating that parallel mutations occur rarely if at all in these simulated viral populations.

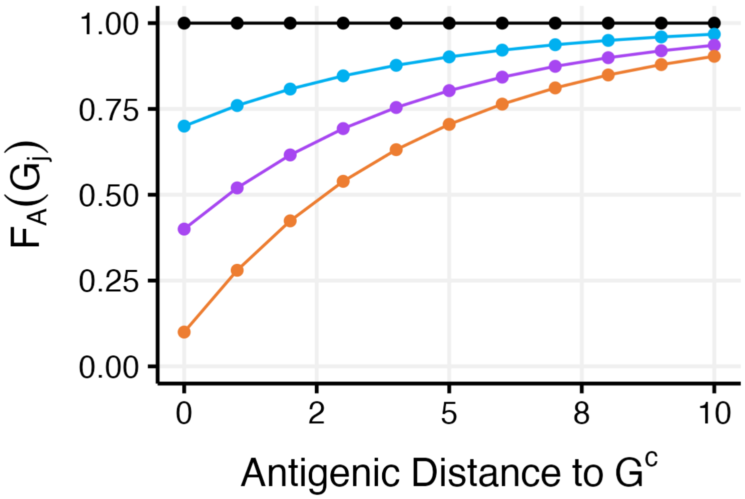

Figure S6. Antigenic fitness parameterizations when considering the impact of different strengths of immune pressure on viral evolution. The plot shows the antigenic fitness of genotypes that are different antigenic distances away from the consensus genotype G^c. The black line shows the antigenic fitness function parameterized with $q = 0$. The light blue line shows F_A with $q = 0.3$, corresponding to immune pressure being low. The purple line shows F_A with $q = 0.6$, corresponding to immune pressure being moderate. The orange line shows F_A with $q = 0.9$, corresponding to immune pressure being strong. The breadth of the immune response in all cases considered here is set to $p = 0.8$.

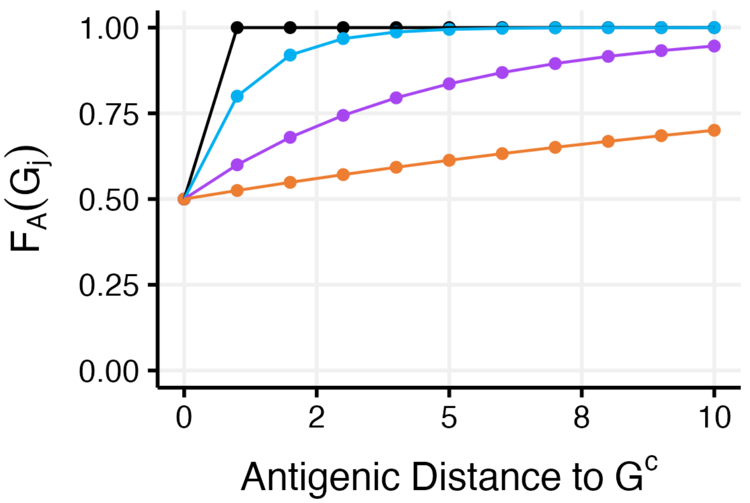

Figure S7. Antigenic fitness parameterizations when considering the impact of different breadths of the immune response on viral evolution. The plot shows the antigenic fitness of genotypes that are different antigenic distances away from the consensus genotype G^c. The black line shows the antigenic fitness function parameterized with $p = 0.0$. The blue line shows F_A with $p = 0.4$. The purple line shows F_A with $p = 0.8$. The orange line shows F_A with $p = 0.95$. The strength of the immune response in all cases considered here is set to $q = 0.5$.

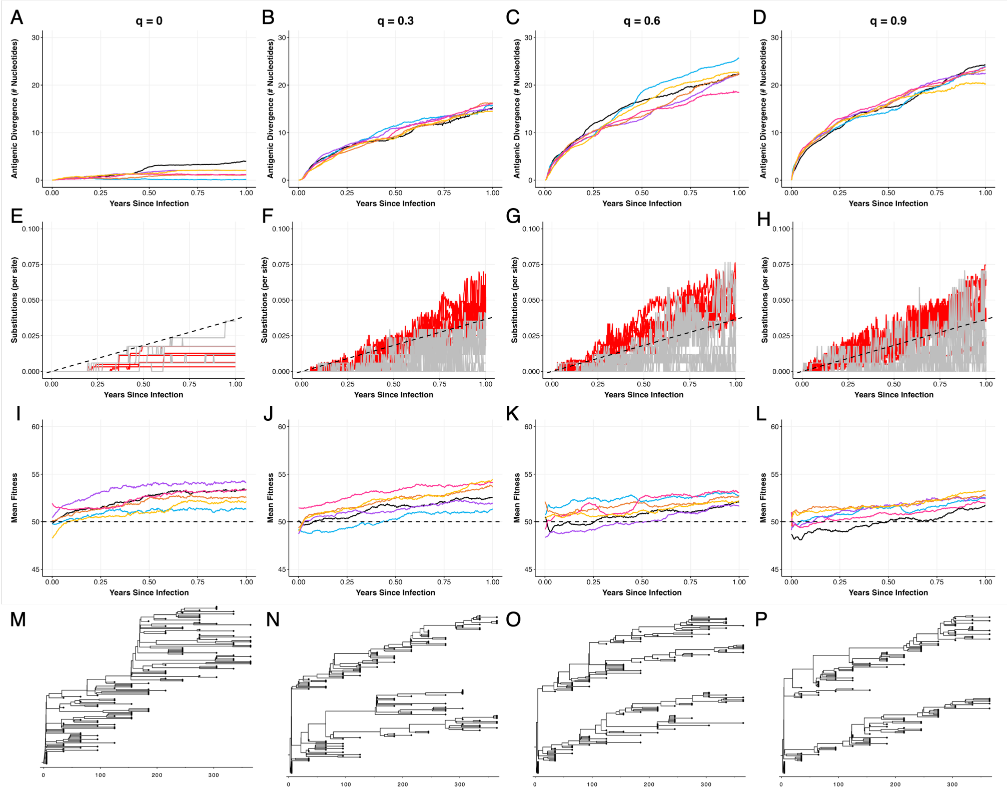

Figure S8. The strength of the immune response impacts patterns of within-host viral evolution in prolonged infections. Here, the viral genome size is $L = 800$, partitioned into $L_P = 534$, $L_{PA} = 96$, $L_A = 0$, and $L_S = 170$. Columns correspond to varying strengths of the immune response: no immune response ($q = 0.0$), low strength ($q = 0.3$), medium strength ($q = 0.6$), and high strength ($q = 0.9$). All simulations had the breadth of the immune response set to $p = 0.8$. (A-D) Extent of antigenic evolution over the course of infection for six independent simulations under each parameterization. Antigenic evolution was calculated as divergence between the consensus genotype at a given time point and the infecting genotype, at the subset of nonsynonymous sites that impacted antigenicity. (E-H) Number of nonsynonymous (red) and synonymous (grey) substitutions per site over the course of infection. Dashed black line shows the expected number of substitutions under neutral evolution. (I-L) Mean viral replicative fitness over the course of infection. The horizontal dashed line shows the expected fitness of the infecting genotype. (M-P) Time-aligned phylogenies for the simulations shown in black in the above panels. The time scale corresponds to the number of days following infection. Simulations were performed using: $N = 5000$, $\mu = 2.5 \times 10^{-5}$ mutations per site per infection cycle, $k = 100$, $c = 0.2$, and $d = 4$ infection cycles per day.

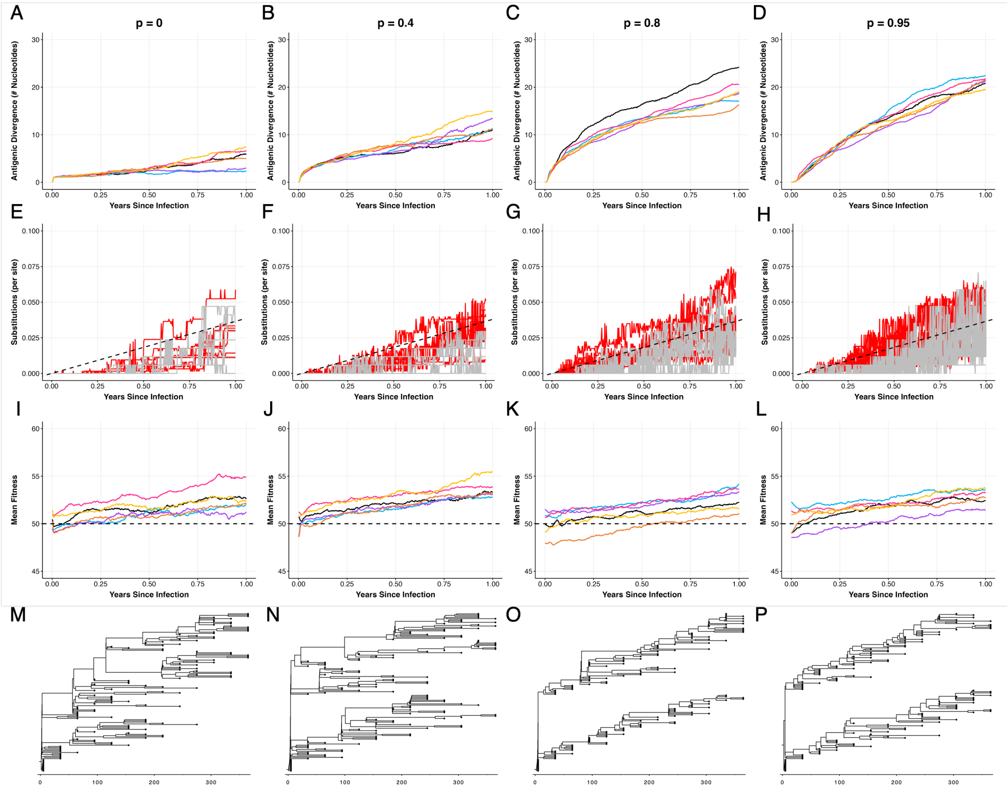

Figure S9. The breadth of the immune response impacts patterns of within-host viral evolution in prolonged infections. Here, the viral genome size is $L = 800$, partitioned into $L_P = 534$, $L_{PA} = 96$, $L_A = 0$, and $L_S = 170$. Columns correspond to varying breadths of the immune response: very narrow breadth ($p = 0.0$), low breadth ($p = 0.4$), medium breadth ($p = 0.8$), and broad breadth ($p = 0.95$). All simulations assumed a moderate strength of immune pressure ($q = 0.5$). (A-D) Extent of antigenic evolution over the course of infection for six independent simulations under each parameterization. (E-H) Number of nonsynonymous (red) and synonymous (grey) substitutions per site over the course of infection. Dashed black line shows the expected number of substitutions under neutral evolution. (I-L) Mean viral replicative fitness over the course of infection. The horizontal dashed line shows the expected fitness of the infecting genotype. (M-P) Time-aligned phylogenies for the simulations shown in black in the above panels. The time scale corresponds to the number of days following infection. Simulations were performed using: $N = 5000$, $\mu = 2.5 \times 10^{-5}$ mutations per site per infection cycle, $k = 100$, $c = 0.2$, and $d = 4$ infection cycles per day.

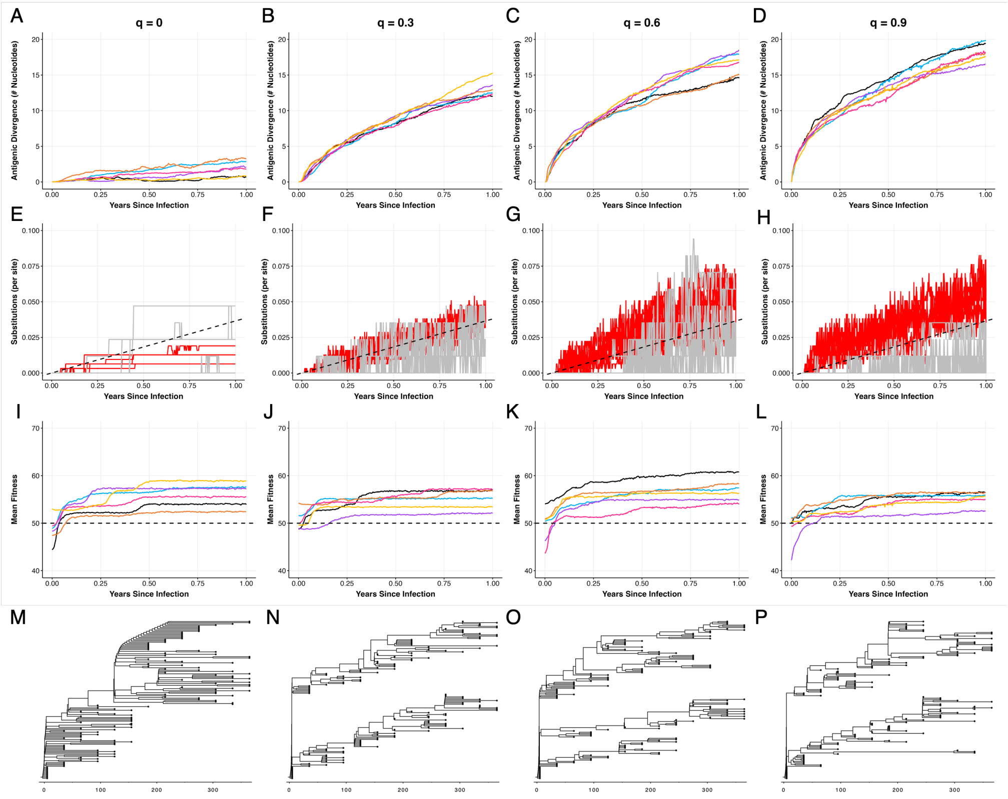

Figure S10. The strength of the immune response impacts patterns of within-host viral evolution in prolonged infections. Here, the viral genome consists of with $L = 400$ sites, partitioned into $L_P = 267$, $L_{PA} = 0$, $L_A = 48$, and $L_S = 170$. Columns correspond to varying strengths of the immune response: no immune response ($q = 0.0$), low strength ($q = 0.3$), medium strength ($q = 0.6$), and high strength ($q = 0.9$). All simulations set the breadth of the immune response to $p = 0.8$. (A-D) Extent of antigenic evolution over the course of infection for six independent simulations under each parameterization. Antigenic evolution was calculated as divergence between the consensus genotype at a given time point and the infecting genotype, at the subset of nonsynonymous sites that impacted antigenicity. (E-H) Number of nonsynonymous (red) and synonymous (grey) substitutions per site over the course of infection. Dashed black line shows the expected number of substitutions under neutral evolution. (I-L) Mean viral replicative fitness over the course of infection. The horizontal dashed line shows the expected fitness of the infecting genotype. (M-P) Time-aligned phylogenies for the simulations shown in black in the above panels. The time scale corresponds to the number of days following infection. Simulations were performed using: $N = 5000$, $\mu = 2.5 \times 10^{-5}$ mutations per site per infection cycle, $k = 100$, $c = 0.2$, and $d = 4$ infection cycles per day.

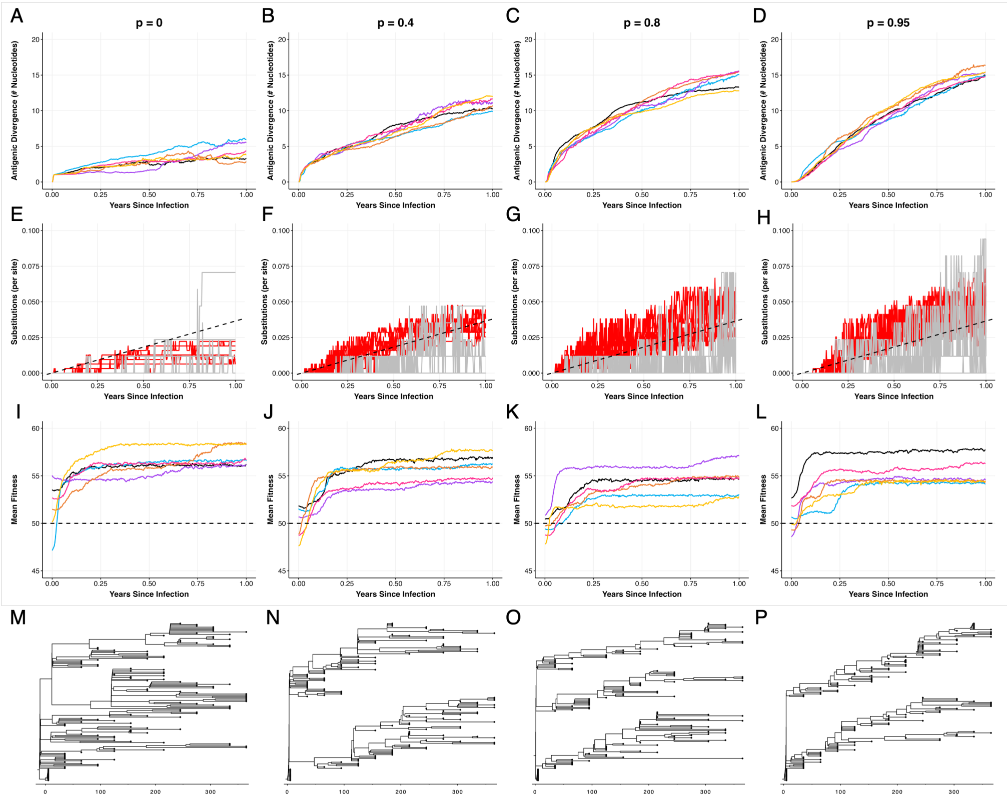

Figure S11. The breadth of the immune response impacts patterns of within-host viral evolution in prolonged infections. Here, the viral genome consists of with $L = 400$, partitioned into $L_P = 267$, $L_{PA} = 0$, $L_A = 48$, and $L_S = 170$. Columns correspond to varying breadths of the immune response: very narrow breadth ($p = 0.0$), low breadth ($p = 0.4$), medium breadth ($p = 0.8$), and broad breadth ($p = 0.95$). All simulations assumed a moderate strength of immune pressure ($q = 0.5$). (A-D) Extent of antigenic evolution over the course of infection for six independent simulations under each parameterization. (E-H) Number of nonsynonymous (red) and synonymous (grey) substitutions per site over the course of infection. Dashed black line shows the expected number of substitutions under neutral evolution. (I-L) Mean viral replicative fitness over the course of infection. The horizontal dashed line shows the expected fitness of the infecting genotype. (M-P) Time-aligned phylogenies for the simulations shown in black in the above panels. The time scale corresponds to the number of days following infection. Simulations were performed using: $N = 5000$, $\mu = 2.5 \times 10^{-5}$ mutations per site per infection cycle, $k = 100$, $c = 0.2$, and $d = 4$ infection cycles per day.

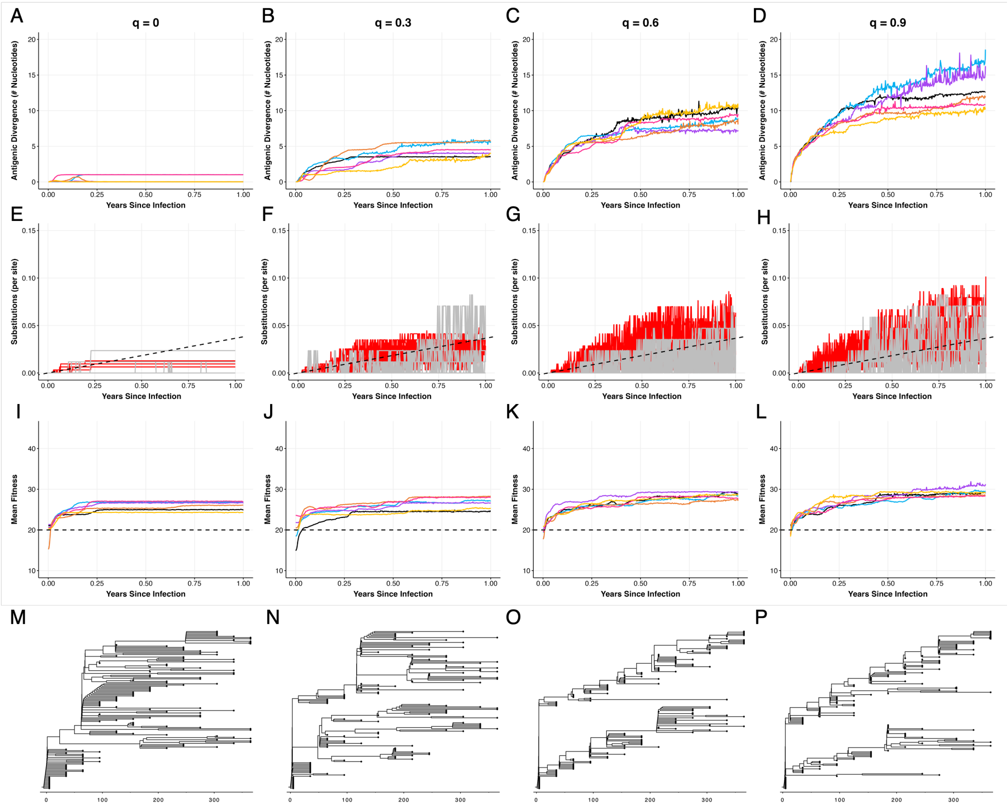

Figure S12. Strong immune pressure facilitates viral adaptation when the infecting genotype is poorly adapted to the host. Columns correspond to varying strengths of the immune response: no immune response ($q = 0.0$) , low strength ($q = 0.3$), medium strength ($q = 0.6$), and high strength ($q = 0.9$). All simulations set the breadth of the immune response to $p = 0.8$. (A-D) Extent of antigenic evolution over the course of infection for six simulations. Antigenic evolution was calculated as divergence between the consensus genotype at a given time point and the infecting genotype, at the subset of sites that impact antigenicity (A and PA sites). (E-H) Number of nonsynonymous (red) and synonymous (grey) substitutions per site over the course of infection. Dashed black line shows the expected number of substitutions under neutral evolution. (I-L) Mean viral replicative fitness over the course of infection. The horizontal dashed line shows the expected fitness of the infecting genotype. (M-P) Time-aligned phylogenies for the simulations shown in black in the above panels. The time scale corresponds to the number of days following infection. Simulations were performed using a viral genome of length $L = 400$, with $L_S = 85$, $L_P = 267$, $L_{PA} = 48$, and $L_A = 0$. Each starting genotype was ∼20% adapted to the host. Other parameters are: $N = 5000$, $\mu = 2.5 \times 10^{-5}$ mutations per site per infection cycle, $k = 100$, $c = 0.2$, and $d = 4$ infection cycles per day.

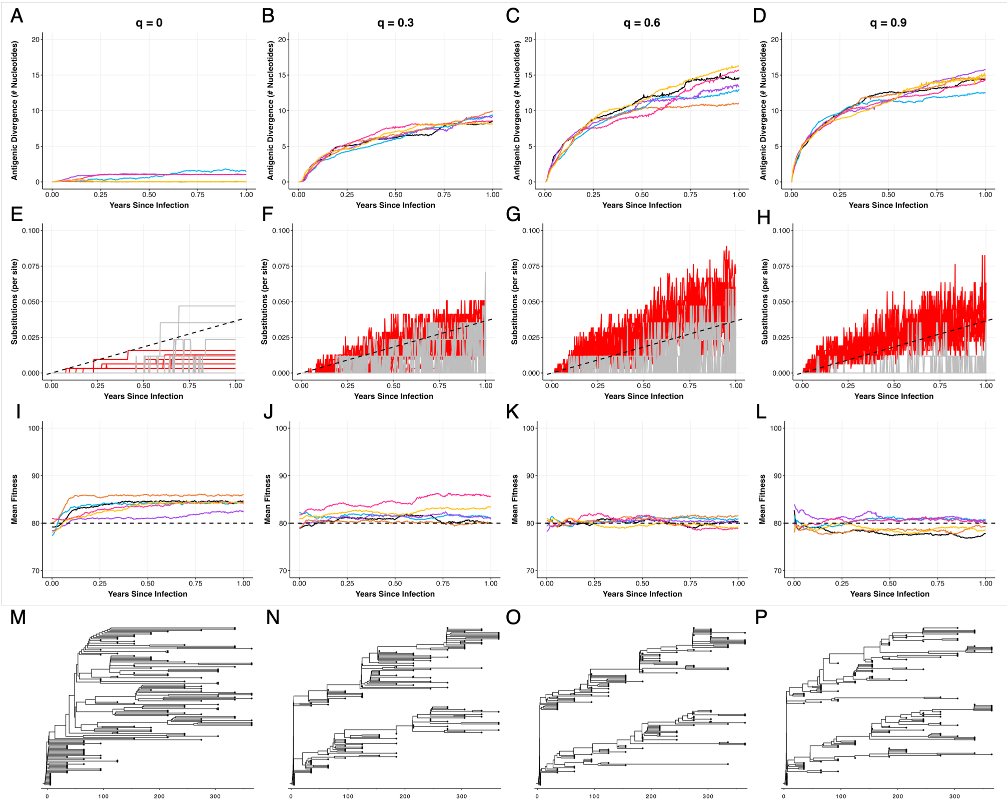

Figure S13. Immune pressure impedes viral adaptation when the infecting genotype is well adapted to the host. Columns correspond to varying strengths of the immune response: no immune response ($q = 0.0$) , low strength ($q = 0.3$), medium strength ($q = 0.6$), and high strength ($q = 0.9$). All simulations set the breadth of the immune response to $p = 0.8$. (A-D) Extent of antigenic evolution over the course of infection for six simulations. Antigenic evolution was calculated as divergence between the consensus genotype at a given time point and the infecting genotype, at the subset of sites that impact antigenicity (A and PA sites). (E-H) Number of nonsynonymous (red) and synonymous (grey) substitutions per site over the course of infection. Dashed black line shows the expected number of substitutions under neutral evolution. (I-L) Mean viral replicative fitness over the course of infection. The horizontal dashed line shows the expected fitness of the infecting genotype. (M-P) Time-aligned phylogenies for the simulations shown in black in the above panels. The time scale corresponds to the number of days following infection. Simulations were performed using a viral genome of length $L = 400$, with $L_S = 85$, $L_P = 267$, $L_{PA} = 48$, and $L_A = 0$. Each starting genotype was $\tilde{8}0\%$ adapted to the host. Other parameters are: $N = 5000$, $\mu = 2.5 \times 10^{-5}$ mutations per site per infection cycle, $k = 100$, $c = 0.2$, and $d = 4$ infection cycles per day.

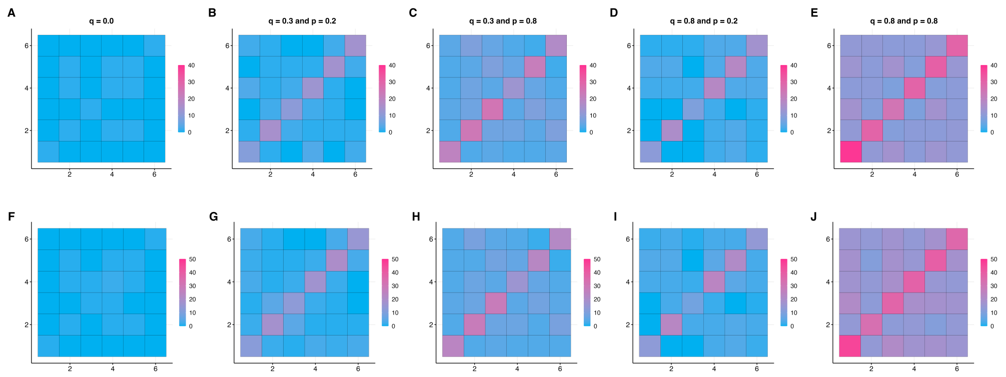

Figure S14. Patterns of parallel mutations from simulations shown in Figure 8 under different definitions of what constitutes a high-frequency mutation. Columns correspond to the different parameterizations of the immune response (depicted in Figure 1H). Column 1: no immune pressure ($q = 0.0$). Column 2: weak immune strength ($q = 0.3$) and narrow immune breadth ($p = 0.2$). Column 3: weak immune strength ($q = 0.3$) and moderate immune breadth ($p = 0.8$). Column 4: strong immune strength ($q = 0.8$) and narrow immune breadth ($p = 0.2$). Column 5: strong immune strength ($q = 0.8$) and moderate immune breadth 4). Infecting genotypes are approximately 50% adapted to the host. Rows correspond to the approach used to identify the set of high-frequency mutations in each individual. (A)-(E) Number of shared, high-frequency nonsynonymous mutations observed across pairs of individuals at time $t = 0.5$ years, reproduced from Figure 8 (panels F-J), for viral populations evolving under different immune pressures. Only mutations that exceeded 20% at time $t = 0.5$ years were considered high-frequency. (F)-(J) Number of shared, high-frequency nonsynonymous mutations observed across pairs of individuals using an alternative approach for identifying the set of high-frequency nonsynonymous mutations in each individual. Here, mutations were considered high-frequency if they reached frequencies of ≥20% at any point in time over the course of infection. The number of identified high-frequency mutations in each individual is, as expected, higher when all mutations that exceeded 20% frequency at any point over the course of infection are considered, rather than only those that exceeded 20% frequency at $t = 0.5$ years following infection. This pattern can be seen by the higher numbers along the diagonal in panels (F)-(J) compared to those in panels (A)-(E). However, results are similar along the off-diagonals, with parallel mutations occurring frequently across individuals in the presence of immune pressure, particularly when immune pressure is strong and immune breath is moderate. Simulations were performed using a viral genome of length $L = 400$, with $L_S = 85$, $L_P = 267$, $L_{PA} = 48$, and $L_A = 0$. Other parameters are: $N = 5000$, $\mu = 2.5 \times 10^{-5}$ mutations per site per infection cycle, $k = 100$, $c = 0.2$, and $d = 4$ infection cycles per day.

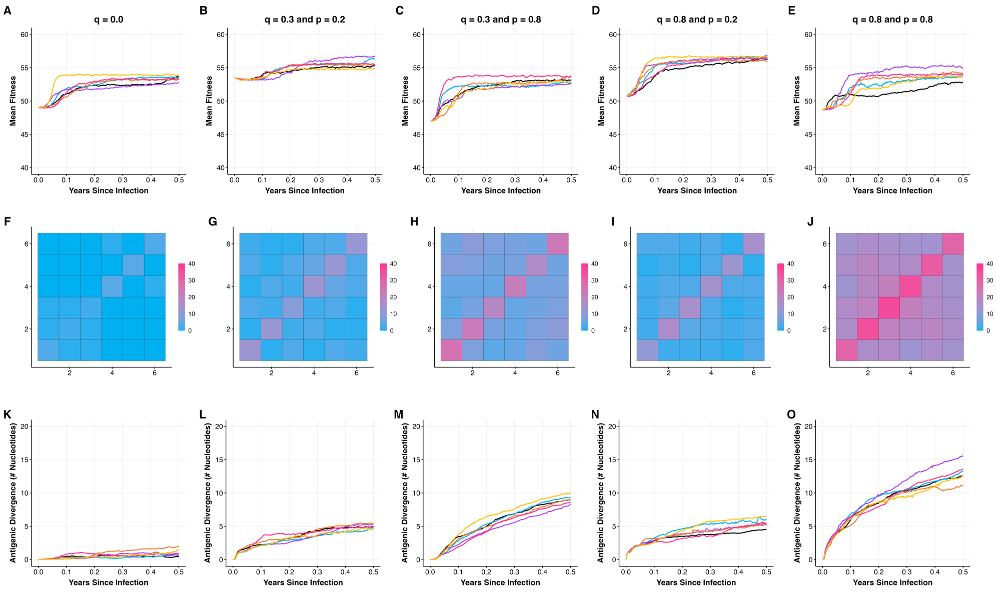

Figure S15. Immune pressure increases the frequency of parallel mutations observed across individuals, regardless of whether the antigenicity-impacting sites are pleiotropic PA sites or only antigenic A sites. Here, the viral genome size is $L = 400$, partitioned into $L_P = 267$, $L_{PA} = 0$, $L_A = 48$, and $L_S = 85$. Columns correspond to varying parameterizations of the immune response that are also depicted in Figure 8. Column 1: no immune pressure ($q = 0.0$). Column 2: weak immune strength ($q = 0.3$) and narrow immune breadth ($p = 0.2$). Column 3: weak immune strength ($q = 0.3$) and moderate immune breadth ($p = 0.8$). Column 4: strong immune strength ($q = 0.8$) and narrow immune breadth ($p = 0.2$). Column 5: strong immune strength ($q = 0.8$) and moderate immune breadth ($p = 0.8$). Infecting genotypes are approximately 50% adapted to the host. (A-E) Changes in mean viral replicative fitness for six viral populations evolving on the same fitness landscape, starting with the same infecting genotype. (F-J) The number of shared mutations across pairs of individuals. Only mutations at nonsynonymous sites that exceeded frequencies of 20% at time $t = 0.5$ years were considered in this calculation. (K-O) Extent of antigenic evolution over the course of infection. All simulations were performed using: $N = 5000$, $\mu = 2.5 \times 10^{-5}$ mutations per site per infection cycle, $k = 100$, $c = 0.2$, and $d = 4$ infection cycles per day. Under each immune response parameterization, the extent of parallel mutations observed in panels F-J are quantitatively similar to those in Figure 5F-J.

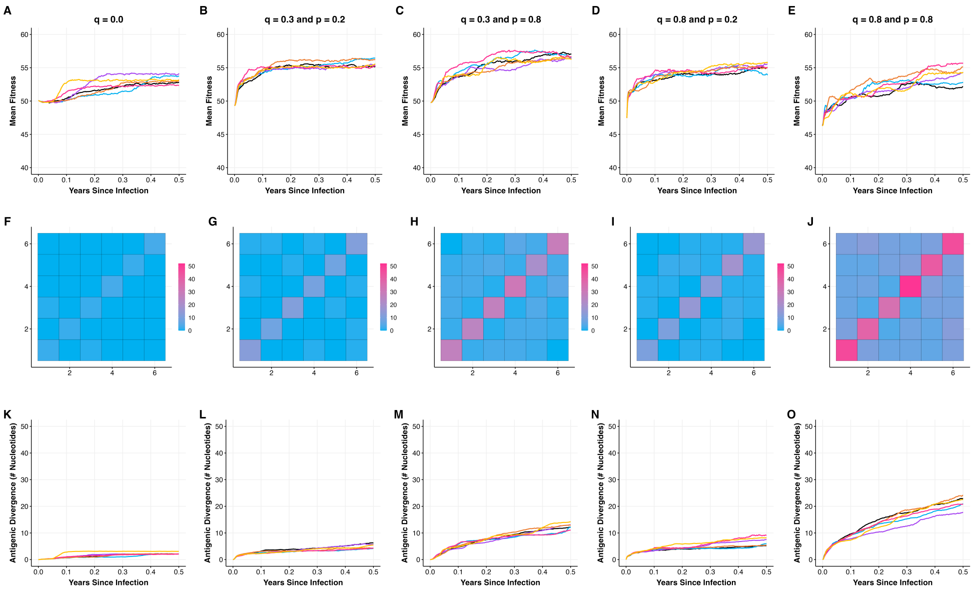

Figure S16. Immune pressure does not substantially increase the frequency of parallel mutations observed across individuals when the number of antigenicity-impacting sites is large. Here, the viral genome size is $L = 400$, partitioned into $L_P = 48$, $L_{PA} = 267$, $L_A = 0$, and $L_S = 85$. Columns correspond to varying parameterizations of the immune response that are also depicted in Figure 8. Column 1: no immune pressure ($q = 0.0$). Column 2: weak immune strength ($q = 0.3$) and narrow immune breadth ($p = 0.2$). Column 3: weak immune strength ($q = 0.3$) and moderate immune breadth ($p = 0.8$). Column 4: strong immune strength ($q = 0.8$) and narrow immune breadth ($p = 0.2$). Column 5: strong immune strength ($q = 0.8$) and moderate immune breadth ($p = 0.8$). Infecting genotypes are approximately 50% adapted to the host. (A-E) Changes in mean viral replicative fitness for six viral populations evolving on the same fitness landscape, starting with the same infecting genotype. (F-J) The number of shared mutations across pairs of individuals. Only mutations at nonsynonymous sites that exceeded frequencies of 20% at time $t = 0.5$ years were considered in this calculation. (K-O) Extent of antigenic evolution over the course of infection. All simulations were performed using: $N = 5000$, $\mu = 2.5 \times 10^{-5}$ mutations per site per infection cycle, $k = 100$, $c = 0.2$, and $d = 4$ infection cycles per day. Under each immune response parameterization, the number of parallel mutations observed in panels F-J is much smaller than those in Figure 5F-J.

References

Nei, M. and Li, W. H. 1979. Mathematical model for studying genetic variation in terms of restriction endonucleases. *Proc. Natl. Acad. Sci. U. S. A.*, 76(10): 5269–5273.
